# Characteristics and prognostic significance of genetic mutations in acute myeloid leukemia based on a targeted next‐generation sequencing technique

**DOI:** 10.1002/cam4.3467

**Published:** 2020-09-24

**Authors:** Rui‐Qi Wang, Chong‐Jian Chen, Yu Jing, Jia‐Yue Qin, Yan Li, Guo‐Feng Chen, Wei Zhou, Yong‐Hui Li, Juan Wang, Da‐Wei Li, Hong‐Mei Zhao, Bian‐Hong Wang, Li‐Li Wang, Hong Wang, Meng‐Zhen Wang, Xiao‐Ning Gao, Li Yu

**Affiliations:** ^1^ Department of Hematology Chinese PLA General Hospital Medical School of Chinese PLA Beijing China; ^2^ Medicine School Nankai University Tianjin China; ^3^ Annoroad Gene Technology Co Beijing Economic‐Technological Development Area Beijing China; ^4^ Beijing Tsinghua Changgung Hospital Changping District Beijing China; ^5^ Department of Hematology‐Oncology Carson International Cancer Center Shenzhen University General Hospital Shenzhen University Health Science Center Shenzhen China

**Keywords:** acute myeloid leukemia, genetic mutations, next‐generation sequencing, prognosis stratification

## Abstract

To explore the characteristics and prognostic significance of genetic mutations in acute myeloid leukemia (AML), we screened the gene mutation profile of 171 previously untreated AML patients using a next‐generation sequencing technique targeting 127 genes with potential prognostic significance. A total of 390 genetic alterations were identified in 149 patients with a frequency of 87.1%. Younger age and high sensitivity to induction chemotherapy were associated with a lower number of mutations. *NPM1* mutation was closely related to *DNMT3A* and *FLT3*‐internal tandem duplication (*FLT3*‐ITD) mutations, but mutually exclusive with *ASXL1* mutation and *CEBPA* ^double mutation^. In univariate analysis, *ASXL1* or *TET2* mutation predicted shorter overall survival (OS) or relapse‐free survival (RFS), *DNMT3A*, *FLT3*‐ITD, or *RUNX1* mutation predicted a higher likelihood of remission‐induction failure, whereas *NRAS* mutation or *CEBPA* ^double mutation^ predicted longer OS. Concurrent *DNMT3A*, *FLT3‐ITD,* and *NPM1* mutations predicted shorter OS. Hypomethylation agents could improve the OS in patients with DNA methylation‐related mutations. According to multivariate analysis, *TET2* mutation was recognized as an independent prognostic factors for RFS. In summary, our study provided a detailed pattern of gene mutations and their prognostic relevance in Chinese AML patients based on targeted next‐generation sequencing screening.

## Graphical abstract

Genetic mutations can predict favorable or unfavorable prognosis of AML patients. Allo stem cell transplantation can provide benefits for AML patients with bad genetic mutations.

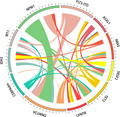

## 1 INTRODUCTION

Acute myeloid leukemia (AML) has the highest mortality among all kinds of hematopoietic neoplasms. Although scientists and doctors have sought for treatments that are more efficient and have less long‐term treatment‐related complications, the remission rate, and long‐term survival of AML are still unfavorable.^1^, ^2^ Even though standard induction therapy based on cytarabine and anthracycline followed by consolidation therapy has been used, 50%‐60% of patients would relapse, and only 40%‐50% of them could achieve long‐term survival.^3^ Furthermore, because AML patients tend to be older with poor‐risk features, a large proportion is unable to tolerate intensive chemotherapy and reach complete remission (CR). Therefore, the current situation regarding the prognosis and therapy of AML remains challenging.^3^

The detection of cytogenetic changes in AML patients at diagnosis for risk stratification and prediction of clinical outcomes is a routine practice,^4^, ^5^, ^6^ but due to the heterogeneity and complexity of AML, there are discrepancies in response to chemotherapy, remission duration, and relapse between individuals, even though they carry the same cytogenetics alterations. More recently, genetic aberrations have been shown to have practical value, and some of these have been applied in National Comprehensive Cancer Network (NCCN) guideline.^6^ Although some of these have been reported to correlate with specific prognoses in clinical studies, some controversy exists surrounding these genetic mutations.^7^, ^8^, ^9^, ^10^, ^11^ For instance, *TET2* mutation had no prognostic significance in AML according to Gaidzik's study, but its unfavorable prognostic impact on AML patients with intermediate‐risk cytogenetics (IR‐AML) was reported by Chou.^12^, ^13^ The impact of *NRAS* mutation on prognosis also changes when the study cohort varies.^5^, ^14^ Therefore, the exact prognostic significance of genetic mutations needs to be further evaluated in a specific cohort of patients.

For the past few decades, cytarabine plus daunomycin, idarubicin, mitoxantrone, or homoharringtonine regimen has been used as standard induction chemotherapy, but relapse is still the noticeable problem during treatment.^8^, ^15^ With the current application of targeted therapy to clinical practice, the treatment of AML is diverse, individualized, and specific.^16^, ^17^, ^18^ Based on the specific genetic mutations, tyrosine kinase inhibitors, *FLT3* inhibitors, and DNA methyltransferase inhibitors can improve survival in AML patients, especially those who are unable to receive conventional chemotherapy.^18^, ^19^, ^20^, ^21^, ^22^, ^23^ Therefore, studies focusing on genetic mutations and targeted agents could provide enormous benefits for AML patients.

A targeted next‐generation sequencing (NGS) technique, a novel kind of applied method for detecting molecular genetic mutations with potential prognostic value, is able to avoid waste of resources and time involved in detecting meaningless mutations.^24^, ^25^ At this point, no comprehensive studies about AML genomics using targeted NGS technique are available for Chinese patients. Therefore, in this study, we explored the characteristics and prognostic significance of genetic mutations using targeted NGS screening in Chinese AML patients.

## 2 METHODS AND MATERIALS

### 2.1 Patient selection

In this study, we enrolled 171 previously untreated AML patients at our unit, and targeted NGS was performed on bone marrow (BM) or peripheral blood (PB) specimens from all enrolled patients from August 2009 to October 2017. The clinical features of the patients, including age, gender, time to diagnosis, origin of disease, karyotype, French‐American‐British (FAB) subtype, hemoglobin level, white blood cell (WBC) count, platelet count, percentage of BM blast, and therapy regimens, are described in Table 1. Secondary AML (s‐AML) was defined as AML with antecedent hematological disorders. Therapy‐related AML (t‐AML) was defined as disease related to prior chemotherapy or toxic exposure history, and de novo AML was defined as disease without antecedent hematological disease or prior cytotoxic therapy. Cytogenetics risk stratification was based on the 2017 European LeukemiaNet risk stratification by genetics.^26^ The study was conducted in accordance with the principles of the Declaration of Helsinki. Signed informed consents were obtained from all patients prior to inclusion in the genetic analyses.

**Table 1:** Clinical characteristics of 171 patients with newly diagnosed AML

Variant	Value
Median age (years old)	48 (19‐88)
Gender ratio (male/female)	93:78
Median hemoglobin (g/L)	81 (26‐145)
Median WBC count (×10^9^/L)	14.1 (1‐333)
Median platelets (×10^9^/L)	42 (5‐621)
BM blasts (%)	54.65 (4‐94)
Origin of disease	
de novo AML	153 (89.5%)
s‐AML	15 (8.8%)
t‐AML	3 (1.7%)
Cytogenetics	
Low‐risk	8 (4.7%)
Intermediate‐risk	125 (73.1%)
High‐risk	20 (11.7%)
Undetermined	18 (10.5%)
FAB subtype	
M1	4 (2.3%)
M2	43 (25.1%)
M4	60 (35.1%)
M5	46 (26.9%)
M6	7 (4.1%)
Undetermined	11 (6.5%)
Response to chemotherapy	
1 cycle CR	78 (45.6%)
2 cycles CR	29 (17.0%)
3 or more cycles CR	5 (2.9%)
NR	18 (10.5%)
Not involved	41 (24.0%)
Patients receiving different therapy	
Conventional chemotherapy alone	84 (49.1%)
HMAs alone	48 (28.1%)
Conventional chemo & HMAs	29 (16.9%)
No chemotherapy	18 (9.9%)
Transplantation	
Allo‐HSCT	53 (31.0%)
Auto‐HSCT	5 (2.9%)
No HSCT	113 (66.1%)
Median follow‐up term (months)	9.5 (0.03‐96.07)

Abbreviations: WBC, white blood cell; BM, bone marrow; s‐AML, secondary AML; t‐AML, therapy‐related AML; CR, complete remission; NR, nonremission; HMAs, hypomethylating agents; Allo‐HSCT, allogenic hematopoietic stem cell transplantation; Auto‐HSCT, autologous hematopoietic stem cell transplantation

### 2.2 Sequencing library construction and NGS through capture

Genomic DNA was extracted from mononuclear cells isolated from the BM or PB samples of each patient at a concentration higher than 10 ng/μL and then underwent a quality control assessment. An Illumina standard DNA library was constructed for high‐throughput sequencing and was then tested by a bioanalyzer 2100 (Agilent Technologies, USA, 50671504) to ensure peak length of approximately 350bp. The hybridization capture chip (Roche NimbleGen) was used to capture 127 specific genes (Supplementary Table S1). After quality control, captured exon libraries were assessed by quantitative PCR with a quantification kit (Kapa Biosystems, USA, NG106‐T1) at a concentration higher than 10 nmol/L.

QPCR was performed in dye method with forward primer (5’TGATACGGCGACCACCGAG‐3’) and reverse primer (5’ AAGCAGAAGACGGCATACGAG‐3’). The following protocol was used: 3 min at 95℃; 35 cycles of 15s at 95℃ and 30s at 60℃. The range of the obtained Ct values was from 15 to 35. And the libraries were sequenced by a Nextseq 550 high‐throughput sequencing instrument (Illumina, USA, 13DW0002). The detailed description of targeted region was shown in Table S2. More information including sequencing metrics was also provided (Table S3).

### 2.3 Data process and bioinformatics analysis

After sequencing, the raw data underwent preprocessing and quality control procedures, which were performed by commercially available tools. Eligible data were aligned to the human reference genome (GRCh37) using BWA algorithms (V0.7.12).^27^ PCR duplication was marked by Picard, whereas the BaseRecalibrator of GATK software (V3.5) ^28^ was used to adjust the results of the data alignment. Based on the paired samples, MuTect2 software (V3.5) was used to gauge single nucleotide variants (SNV) and inversion and deletion (indel) variants.^29^ In addition, *FLT3*‐internal tandem duplication (*FLT3*‐ITD) mutation and *MLL* partial tandem duplication (*MLL*‐PTD) identification were accomplished by methods developed in‐house. All the results were annotated with ANNOVAR (V0722).^30^ Variant allele frequency (VAF) was calculated as the number of the variant reads divided by the total number of reads for the mutation position. VAF ≥ 1% retained for further analysis.

### 2.4 Statistical analysis

Overall survival (OS) was defined as the period from diagnosis to the end point, such as death or the last time of follow‐up. Relapse‐free survival (RFS) was defined as the period from first complete remission to relapse, death or the last time of follow‐up. Sensitive response to chemotherapy was defined as reaching CR status through one or two cycles of induction of chemotherapy, whereas refractory response to chemotherapy was defined as primary induction failure or reaching CR through three or more cycles of induction of chemotherapy. The CR rate was calculated after two cycles of induction chemotherapy.

All the statistical processes were performed using SPSS software (version 19.0). Shapiro‐Wilk test was used to explore whether the values in each group had normal distribution because the number of samples was far below 5000. ANOVA was used to calculate the difference in mutation number among groups if the values in each group had normal distribution; if not, nonparametric tests (＞2 groups, Kruskal‐Wallis test; =2 groups, Mann‐Whitney U test) were used. Two‐sample Student's t‐test was used to compare continuous variables (age, hemoglobin level, WBC count, platelet count, and percentage of BM blast) in different groups differed by mutational status of genes. The chi‐squared test and Fisher's exact test were used to compare the differences in response to chemotherapy and other categorical variables (gender, cytogenetic‐risk, origin of disease, and FAB subtype) with specific mutational status. OS and RFS were calculated using Kaplan‐Meier analysis and the COX proportional hazard regression analysis was used for multivariate survival analysis. A two‐sided *p* value was considered to indicate statistical significance if *P* < .05.

## 3 RESULTS

### 3.1 Spectrum of genetic mutations in AML

**Figure 1:**
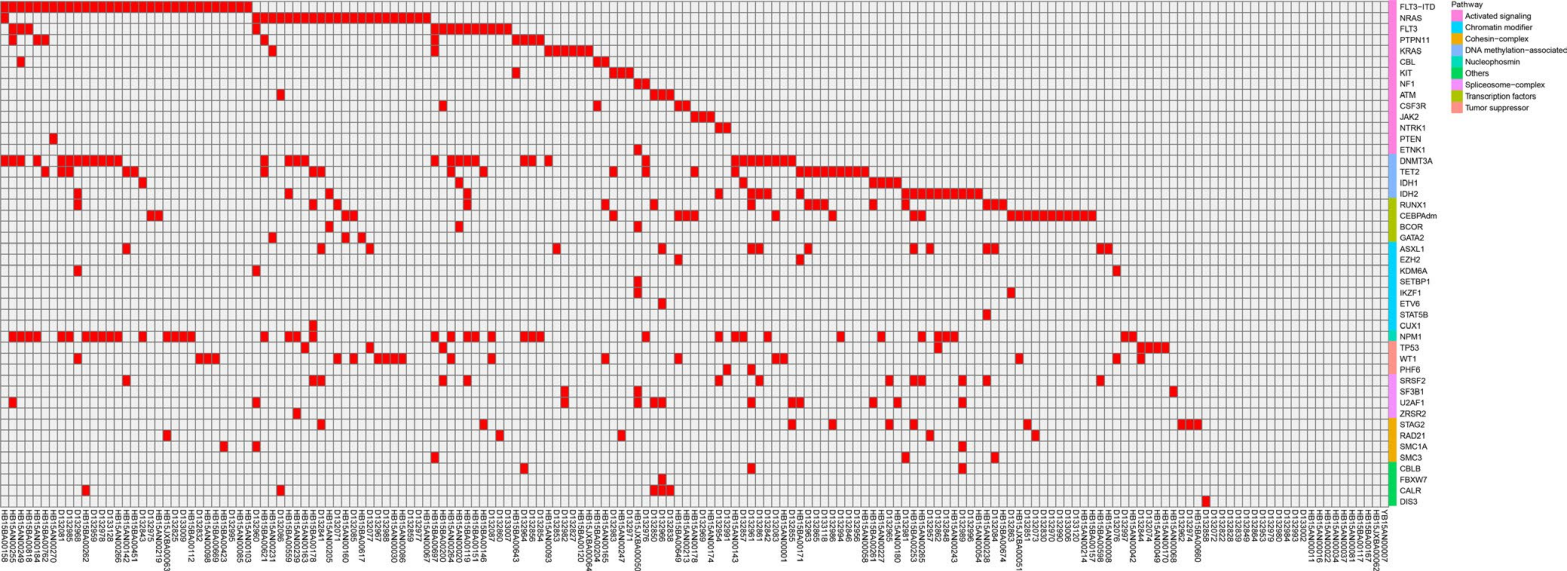
The landscape of genetic mutations classified into nine functional clusters (Activated signaling genes, chromatin modifier, cohesion‐complex, DNA methylation‐associated genes, mucleophasmin, spliceosome‐complex, transcription factors, tumor suppressor, and others) in adult acute myeloid leukemia (AML)

The clinical characteristics of the 171 patients with newly diagnosed AML are shown in Table 1. As shown in Figure 1, a total of 390 genetic alterations were identified in 149 patients among 171 cases with a frequency of 87.1%. There were 22 patients without detectable mutations, whereas 112 patients were found to have at least two mutations. All variants detected in our research were exhibited in Tables S4 and S5. There were eight genes mutated in more than 10% of patients. The most commonly mutated gene was *NPM1* (24.0%), followed by *DNMT3A* (19.3%), *FLT3*‐ITD (18.1%), *CEBPA* ^double mutation^ (*CEBPA* ^dm^ 13.5%), *NRAS* (13.5%), *TET2* (13.5%), *IDH2* (11.1%), and *WT1* (11.1%) (Figure S1).

### 3.2 Co‐occurrence and mutual exclusivity pattern of genetic mutations in AML

**Figure 2:**
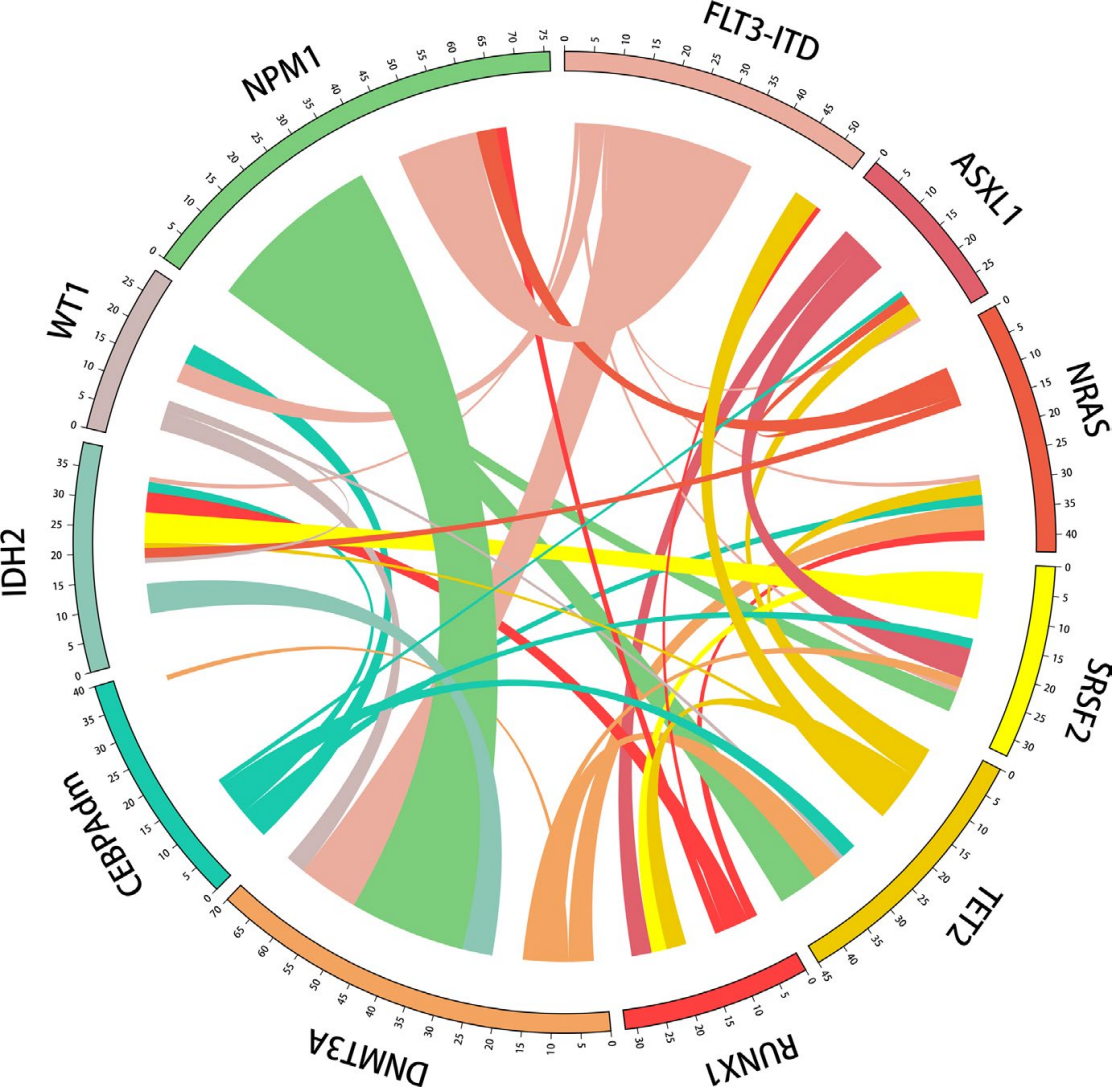
Circos diagram representing the co‐occurrence of gene mutations in *DNMT3A*, *FLT3*‐ITD, *NRAS*, *TET2*, *CEBPAdm*, *IDH2*, *WT1*, *RUNX1*, *ASXL1*, *SRSF2,* and *NPM1*. The length of the arc represents the frequency of mutations in the first gene, and the width of the ribbon represents the percentage of patients carrying a mutation in the second gene

We explored the co‐occurrence and mutual exclusivity pattern of genetic mutations with frequencies higher than 10% in the entire cohort of AML patients. Although the frequencies of *ASXL1*, *RUNX1,* and *SRSF2* are lower than 10% in the total cohort, we still took them into consideration cause they have been reported to strongly correlate with AML prognosis. As shown in Figure 2, *NPM1* mutation was significantly correlated with *DNMT3A* (*p*＜0.001) and *FLT3*‐ITD (*p*＜0.001) mutations, but was mutually exclusive with *CEBPA* ^dm^ (*P* = .017) and *ASXL1* mutations (*P* = .024). Besides, *FLT3*‐ITD is also accompanied with *DNMT3A* mutations (*P* = .005). Mutant *ASXL1* was significantly correlated with *SRSF2* (*p*＜0.001) and *RUNX1* mutations (*P* = .023) (Figure S2).

### 3.3 Association of genetic mutations with baseline clinical features of AML patients

We studied the relationships of genetic mutations with baseline clinical features of AML patients, including age, gender, cytogenetic risk, BM blast percentage, FAB subtype, and hemoglobin, WBC and platelet counts at diagnosis (Table S6). *FLT3*‐ITD was associated with a higher count of WBCs at diagnosis (*P* = .004) and tended to occur in female patients (*P* = .002). *RUNX1*, *DNMT3A,* and *ASXL1* mutations were more likely to exist in older patients (*P* = .038, *P* = .013, and *P* = .022, respectively). In addition, a higher mutation rate of *ASXL1* was found in patients with lower levels of hemoglobin (*P* = .018), and *CEBPA* ^dm^ predominated in those with higher levels of hemoglobin (*P* = .003). Notably, other gene mutations were not found to be significantly associated with the above clinical features.

We then studied the relationship between the numbers of genetic mutations and clinical characteristics including age, FAB classification, origin of disease, cytogenetic risk, and response to chemotherapy (Table S7). As stated before, we made normality test for each comparison and found that at least one group did not have normal distribution. Therefore, Kruskal‐Wallis test and Mann‐Whitney U test were used to complete multiple comparison. With regard to age, we divided all patients into three groups (18‐39, 40‐59,≥60) and found that the number of genetic mutations in patients aging from 18 to 39 was significantly lower than that in patients aging from 40 to 59 (*P* = .043) and those over 60 years old (*P = *.016, Figure S3A). With regard to response to chemotherapy, we found sensitive patients had a significantly lower number of genetic mutations than those with refractory disease (*P = *.031, Figure S3B). Stratified by FAB subtype, patients in M4 subtype carried a significantly higher number of genetic mutations than those in M2 or M5 subtype (*P = *.004 and 0.010, respectively; Figure S3C). But considering cytogenetic risk and origin of disease, respectively, we found no significant difference in the number of mutations among each subgroup (Supplementary Figure SD‐E).

### 3.4 Genetic mutation predicted rate of complete remission

We evaluated the impact of single genetic mutation on the rate of CR in the total cohort of AML patients and different subgroups according to the origin of disease and cytogenetic risks. As for the following survival analysis, we still make the same exclusion. In the total cohort, the rate of CR was significantly lower in patients with *FLT3*‐ITD (60.9% *vs*. 86.1%, *P = *.008) or mutant *DNMT3A* (64.0% *vs*. 85.8%, *P = *.019) than in those without the above gene mutations. In patients with IR‐AML, besides those with mutations in *FLT3*‐ITD (60.0% *vs*. 84.5%, *P = *.026) or *DNMT3A* (59.1% *vs*. 85.4%, *P = *.014), patients with mutant *RUNX1* (42.9% *vs*. 82.5%, *P = *.029) had a significantly lower rate of CR than those with wild type. In patients with de novo AML, *FLT3*‐ITD (55.0% *vs*. 87.7%, *P* = .002) or *DNMT3A* (62.5% *vs*. 87.3%, *P* = .013) mutations remained an unfavorable factor predicting a lower rate of CR compared with the absence of these gene mutations (Table S8).

### 3.5 Genetic mutation predicted survival

We evaluated the prognostic significance of genetic mutations classified by functional clustering in aspects of age, origin of disease, and cytogenetic risk. In our study, mutant *TET2*, *DNMT3A*, *ASXL1*, and *FLT3*‐ITD showed inferior impacts on survival of AML patients, whereas *NRAS* mutation and *CEBPAdm* exhibited the opposite influence.

**Figure 3:**
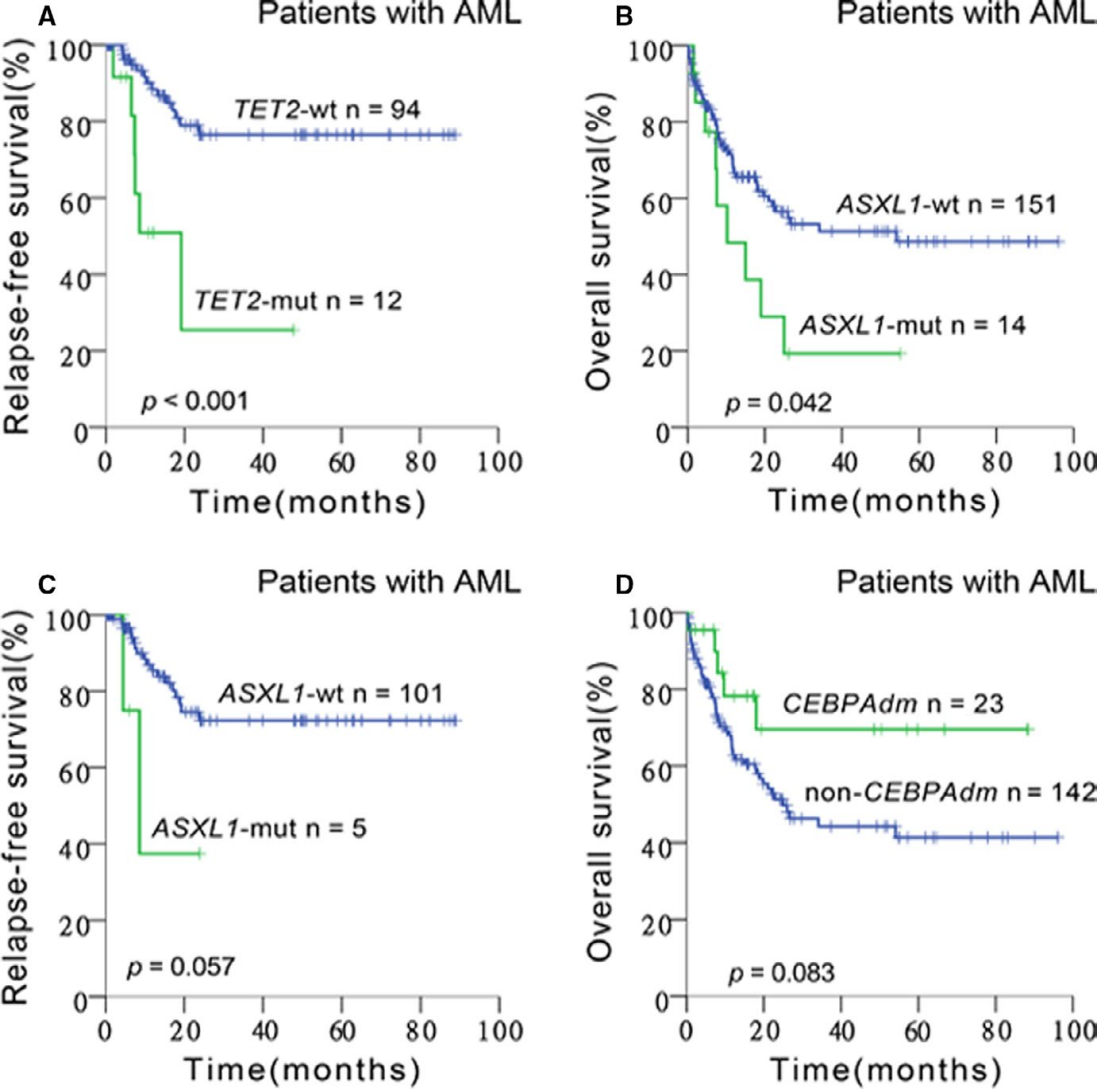
Kaplan‐Meier curves for survival of patients with or without specific genetic mutations in AML. The green and blue lines represent the survival of patients with or without mutations, respectively. (A) RFS for patients with or without *TET2* mutations (*P* < .001). (B) OS for patients with or without *ASXL1* mutations (*P* = .042). (C) RFS for patients with or without *ASXL1* mutations (*P* = .057). (D) OS for patients with or without *CEBPAdm* (*P* = .083). RFS, relapse‐free survival; OS, overall survival

*TET2* and *DNMT3A* belong to the DNA methylation‐associated gene family. In this study, although mutant *TET2* did not influence OS in the total cohort, *TET2* mutations were associated with an adverse RFS in the total cohort (2‐year RFS: 25.5% *vs* 76.5%, *P* < .001; Figure 3A). Regarding cytogenetic risk, we found that mutant *TET2* had an inferior impact on RFS in IR‐AML (2‐year RFS: 0% *vs* 75.2%, *P* = .004) as well as in CN‐AML (2‐year RFS: 0% *vs* 83.9%, *P* < .001) (Figure S4A‐B). Mutant *DNMT3A* did not display any impact on survival when the study cohort included all the patients, but a significantly unfavorable impact on OS of mutant *DNMT3A* could be observed in CN‐AML (2‐year OS: 44.3% *vs* 60.9%, *P = *.024; Figure S4C).

The *ASXL1* gene is a member of a chromatin modifier gene family, predicting significantly unfavorable OS (2‐year OS: 29.0% *vs* 57.7%, *P = *.042) and a trend of inferior RFS (2‐year RFS: 0% *vs* 72.3%, *P = *.057) in the total cohort of patients (Figure 3B‐C). Mutant *ASXL1* was also related to an unfavorable OS in patients under the age of 60 years (2‐year OS: 33.3% *vs* 64.0%, *P* = .015) or with IR‐AML (2‐year OS: 26.3% *vs* 55.8%, *P = *.025; Figure S4D‐E).

*FLT3*‐ITD and *NRAS* belong to the activated signaling gene family. In this study, we did not find a prognostic impact of *FLT3*‐ITD on the survival of AML. However, when we restricted the study cohort to within patients not receiving allogeneic hematopoietic stem cell transplantation (allo‐HSCT), the patients with *FLT3*‐ITD had a significantly inferior OS compared to those with wild‐type *FLT3* (2‐year OS: 0% *vs* 38.0%, *P = *.014) (Figure S4F). Mutant *NRAS* was associated with a superior OS both in patients with IR‐AML (2‐year OS: 76.4% *vs* 48.7%, *P = *.014) (Figure S4G) and in CN‐AML (2‐year OS: 100% *vs* 51.0%, *P = *.010) (Figure S4H).

*CEBPα* belongs to a transcription factor gene family. In this study, *CEBPA* ^dm^ conveyed a better trend of OS in AML (2‐year OS: 70.5% *vs* 52.7%, *P = *.083; Figure 3D). If we restricted the study cohort to patients under 60 years of age, *CEBPA* ^dm^ was associated with a significantly prolonged OS (2‐year OS: 80.8% *vs* 58.8%, *P = *.041; Figure S4I). With respect to RFS, *CEBPA* ^dm^ has no impact on RFS of AML patients in the total cohort in our study (2‐year RFS: 80.7% *vs* 70.4%, *P* = .734).

### 3.6 Concurrent mutations of *DNMT3A*, *FLT3*‐ITD, and *NPM1* predicted inferior survival

**Figure 4:**
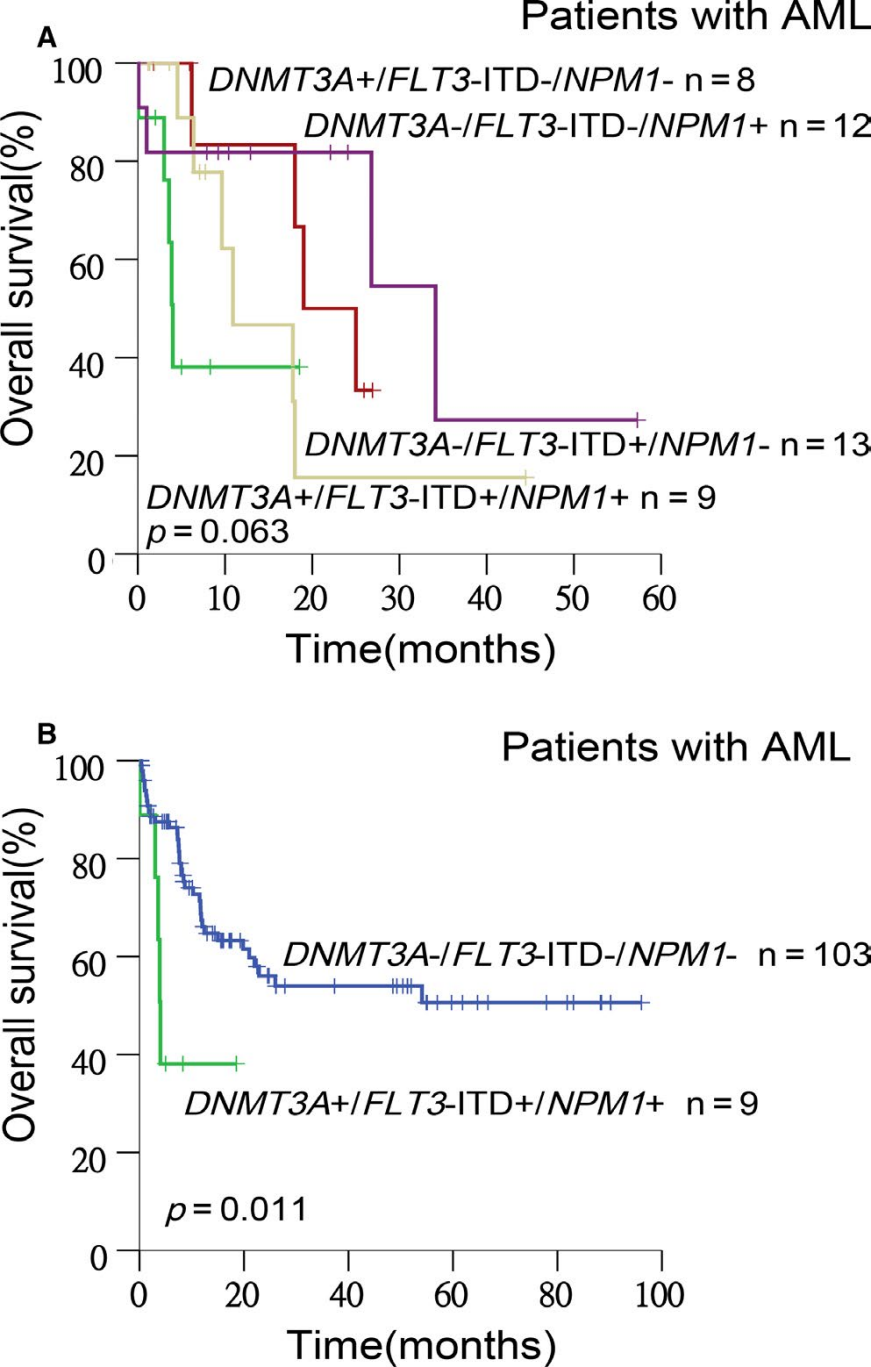
Kaplan‐Meier curves for OS stratified by the mutation status determined by *DNMT3A*, *FLT3,* and *NPM1* genes. Lines in different colors represent the survival of patients in different groups, respectively. (A) A difference in OS was shown among AML*^DNMT3A^* ^+/^ *^FLT3^* ^‐ITD+/^ *^NPM1^* ^+^(the green line), AML*^DNMT3A^* ^‐/^ *^FLT3^* ^‐ITD+/^ *^NPM1^* ^‐^(the yellow line), AML*^DNMT3A^* ^+/^ *^FLT3^* ^‐ITD‐/^ *^NPM1^* ^‐^(the red line), and AML*^DNMT3A^* ^‐/^ *^FLT3^* ^‐ITD‐/^ *^NPM1^* ^+^(the purple line) (*P* = .063). (B) AML*^DNMT3A^* ^+/^ *^FLT3^* ^‐ITD+/^ *^NPM1^* ^+^(the green line) had significantly reduced OS compared with AML*^DNMT3A^* ^‐/^ *^FLT3^* ^‐ITD‐/^ *^NPM1^* ^‐^(the blue line) (*P* = .011)

Since each of *DNMT3A*, *FLT3*‐ITD, and *NPM1* genes predicted relatively higher incidences of mutation compared to other genes in the total cohort and significantly co‐occurred with each other, we explored their comprehensive prognostic meanings in AML. At first, we compared patients harboring these three mutations with those harboring any two of these mutations. Among AML*^DNMT3A^* ^+/^ *^FLT3^* ^‐ITD+/^ *^NPM1^* ^+^, AML*^DNMT3A^* ^+/^ *^NPM1^* ^+/^ *^FLT3^* ^‐ITD‐^, AML*^DNMT3A^* ^+/^ *^FLT3^* ^‐ITD+/^ *^NPM1^* ^‐^, and AML*^FLT3^* ^‐ITD+/^ *^NPM1^* ^+/^ *^DNMT3A^* ^‐^, there was no significant difference in OS. Then we compared AML*^DNMT3A^* ^+/^ *^FLT3^* ^‐ITD+/^ *^NPM1^* ^+^ with those harboring any one of these mutations (AML*^DNMT3A^* ^‐/^ *^FLT3^* ^‐ITD+/^ *^NPM1^* ^‐^, AML*^DNMT3A^* ^+/^ *^FLT3^* ^‐ITD‐/^ *^NPM1^* ^‐^, and AML*^DNMT3A^* ^‐/^ *^FLT3^* ^‐ITD‐/^ *^NPM1^* ^+^) and found a difference in OS (2‐year OS: 0% *vs* 15.6% *vs* 50.0% *vs* 81.8%, *P = *.063), with AML*^DNMT3A^* ^‐/^ *^FLT3^* ^‐ITD‐/^ *^NPM1^* ^+^ having the best OS (Figure 4A). AML*^DNMT3A^* ^+/^ *^FLT3^* ^‐ITD+/^ *^NPM1^* ^+^ still conveyed a significantly reduced OS (2‐year OS: 0% *vs* 56.1%, *P = *.011) compared with genotypes without any of these three mutations (Figure 4B).

### 3.7 The effect of a hypomethylating agent on DNA methylation‐associated genetic mutation

**Figure 5:**
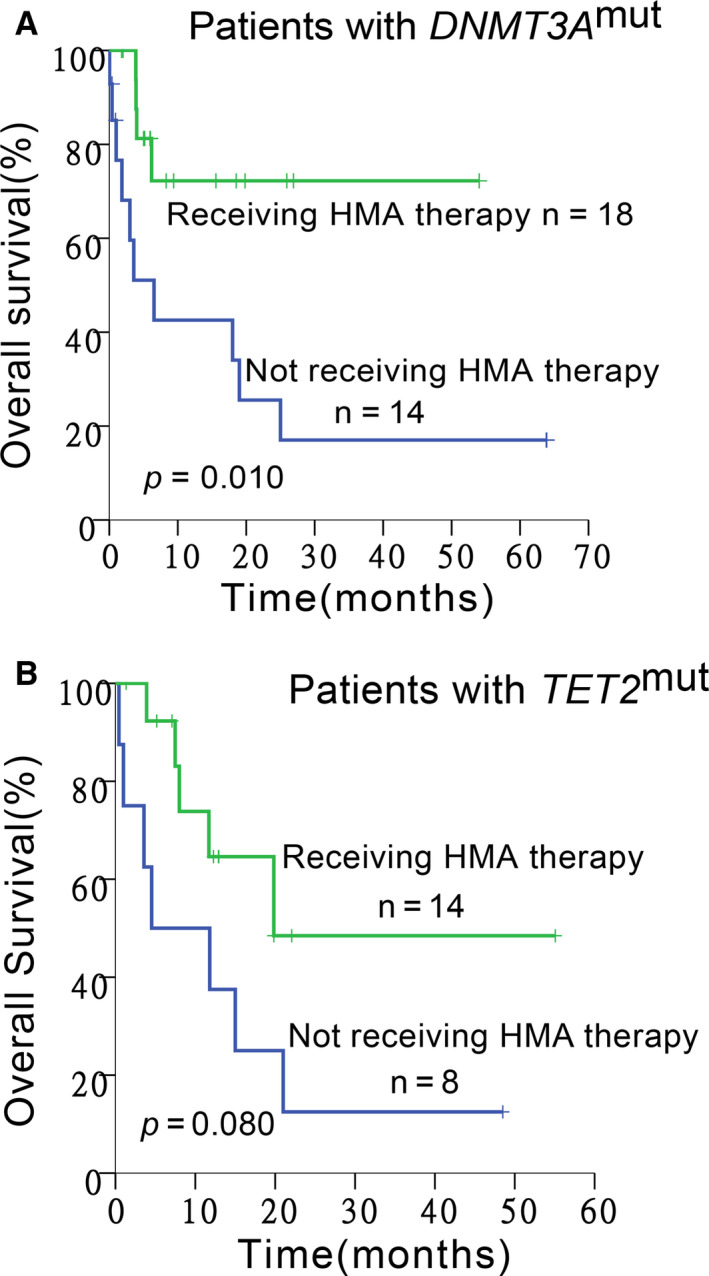
Kaplan‐Meier curves for OS of AML patients with the DNA methylation‐associated genetic mutation stratified by the situation of using HMA. In *DNMT3A*‐mutated (A) or *TET2*‐mutated (B) group, patients receiving HMA therapy (the green lines) had better OS than those never receiving HMA (the blue lines) (*P* = .010 and 0.080, respectively). HMAs, hypomethylating agents

Based on the above observations that DNA methylation‐associated genetic mutation is related to unfavorable survival, there have been many kinds of treatments used in AML patients. Among patients harboring *DNMT3A* mutations, some had ever been given hypomethylating agents (HMAs), whereas others had never been given HMAs, so we further explored the exact benefit of HMAs in AML patients carrying such types of mutations. We found that patients with mutant *DNMT3A* receiving HMA treatment displayed a significantly better OS than those not receiving HMA treatment (2‐year OS: 72.2% *vs* 25.5%, *P = *.010). Patients with mutant *TET2* also displayed a trend of better OS after HMA treatment, although the superiority wasn't statistically significant (2‐year OS: 48.5% *vs* 12.5%, *P = *.080; Figure 5A‐B). But HMAs did not prolong the RFS of AML patients with *TET2* (2‐year RFS: 0 *vs* not reach, *P* = .206) or *DNMT3A* mutation (2‐year RFS: 66.7% *vs* 50.0%, *P* = .305), which needs to be explored further due to the small sample size of specific cohort.

### 3.8 Multivariate analysis of survival of AML patients

Variables including age, gender, origin of disease, cytogenetic risk, FAB subtype, WBC count, treatment response, HMA treatment, high‐dose cytarabine treatment, transplantation, and genetic mutations were included in the multivariate analysis. For OS, allo‐HSCT was an independent favorable factors, whereas a WBC count of more than 100 × 10^9^/L and failure to achieve a CR after two cycles of induction chemotherapy were unfavorable factors. For RFS, with the exception of allo‐HSCT, *TET2* mutation and s‐AML were independent unfavorable factors (Table 2). Notably, we confirmed the favorable effect of HMA on DNA methylation‐associated genetic mutations, but in multivariate analysis, the use of HMAs was not an independent prognostic factor (Table S9).

**Table 2:** Multivariate analysis for survival of newly diagnosed AML patients

Factors	HR (95%CI)	*p* value
OS		
Allo‐HSCT (yes *vs* no)	0.337 (0.157‐0.724)	.005
WBC count (>100 × 10^9^/L *vs* ≤ 100×10^9^/L)	2.634 (1.066‐6.509)	.036
Number of cycles to CR (≥3 cycles *vs* ≤ 2 cycles)	5.313 (2.733‐10.328)	<.001
RFS		
Origin of disease (s‐AML *vs* de novo AML)	24.405 (2.373‐250.944)	.007
Allo‐HSCT (yes *vs* no)	0.234 (0.077‐0.704)	.010
*TET2* mutation (yes *vs* no)	7.569 (2.308‐24.818)	.001

Abbreviations: OS, overall survival; Allo‐HSCT, allogenic hematopoietic stem cell transplantation; WBC, white blood cell; CR, complete remission; RFS, relapse‐free survival; s‐AML, secondary AML.

## 4 DISCUSSION

AML is a kind of hematopoietic neoplasm with high heterogeneity, which is reflected by complex cytogenetic changes and molecular genetic aberrancy.^31^ AML patients were divided into three risk groups based on the NCCN guideline: low‐risk cytogenetics, intermediate‐risk cytogenetics and high‐risk cytogenetics.^6^ Moreover, molecular genetic markers also play a critical role in judging risk and prognosis assessment.^32^, ^33^, ^34^, ^35^ Therefore, testing genetic markers at diagnosis and performing precise risk stratification for therapeutic guidance and maximum efficiency is highly practical.

In our study, the five most common mutated genes were successively *NPM1*, *DNMT3A*, *FLT3*‐ITD, *NRAS,* and *CEBPA^dm^*, nearly in accordance with the result of Metzeler's study in which the top five mutated genes were *FLT3*, *NPM1*, *DNMT3A*, *NRAS,* and *RUNX1*.^36^ No significant difference in frequencies of mutations was observed.

According to the multistage hypothesis of tumor evolution, various mutations can contribute to the development of tumor, accumulate and play roles in different stages, leading to the growing number of genetic mutations. In this study, we explored the association of the number of genetic mutations with clinical characteristics and found that the number of gene mutations was significantly lower in AML patients aging from 18 to 39 years old than in those over 40 years old within adult patients, suggesting that the number of gene mutation in each AML patient increased with age. With respect to the response to chemotherapy, the phenomenon of sensitive patients having significantly fewer genetic mutations than refractory patients exhibiting a strong relationship between mutation number and chemotherapeutic effect indicates that patients with more mutations tend to be more difficult to cure completely; thus, this type of patient needs to be actively scheduled for allogeneic hematopoietic stem cell transplantation.

In the total cohort, *NPM1* mutation was significantly correlated with *FLT3*‐ITD and *DNMT3A* mutations, but was mutually exclusive with *ASXL1* mutation and *CEBPA* ^dm^ in our study, which was in accordance with other reports and suggested that *NPM1* mutation might share a similar mechanism carcinogenesis with *ASXL1* mutation and *CEBPA*.^1^, ^37^, ^38^ As reported previously, *TET2* and *IDH* mutations are mutually exclusive with each other,^39^ but we did not find such a phenomenon in our study.

According to our results, *ASXL1* and *TET2* mutations, both belonging to epigenetic‐related genes, significantly predicted inferior OS and RFS. There is still controversy over *TET2* mutation regarding its prognostic impact on AML in different studies.^40^, ^41^ We confirmed that *TET2* mutation had an unfavorable impact on RFS not OS in AML, not only in the total cohort, but also in the IR‐AML and CN‐AML subgroups, suggesting that the unfavorable impact of *TET2* mutations was independent of cytogenetics. To exclude the effect of allo‐HSCT, we used chi‐squared test and found that whether patients received allo‐HSCT or not had no association with whether patients carried *TET2* mutations or not. So the result wasn't an effect of allo‐HSCT (Table S10). Besides, in multivariate analysis, the impact of *TET2* mutation on RFS remained significant; thus, *TET2* mutation might be correlated with relapse of leukemia. Also, its occurrence is an early event in the course from normal hematopoiesis to AML, which plays an important role in diagnosis, risk stratification, and therapy guidance.^42^ Furthermore, although *DNMT3A* mutation did not show any prognostic impact in the total cohort of AML patients, it was associated with significantly unfavorable OS in CN‐AML subgroup. Notably, *ASXL1*, *TET2,* and *DNMT3A* genes are all epigenetic‐related genes, so our results suggested their roles in the occurrence and progression of AML.

We found that in addition to *DNMT3A*, *FLT3*‐ITD, and *RUNX1* mutations were also related to chemotherapy resistance. There was no difference in survival between AML patients with *FLT3*‐ITD and patients with wild‐type *FLT3*, but in cases not receiving allo‐HSCT, *FLT3*‐ITD was associated with a significantly reduced OS, illustrating that allo‐HSCT might overcome the adverse effect of *FLT3*‐ITD on the survival of AML patients.

*NPM1* mutation, a widely reported favorable prognostic factor of survival in AML,^5^, ^36^, ^42^ had no association with prognosis in AML in our study. In light of the fact that AML*^DNMT3A^* ^‐/^ *^FLT3^* ^‐ITD‐/^ *^NPM1^* ^+^ had the best OS compared with AML*^DNMT3A^* ^+/^ *^NPM1^* ^+/^ *^FLT3^* ^‐ITD‐^, AML*^DNMT3A^* ^+/^ *^FLT3^* ^‐ITD+/^ *^NPM1^* ^‐^, and AML*^DNMT3A^* ^+/^ *^FLT3^* ^‐ITD+/^ *^NPM1^* ^+^, we hypothesized that the concomitant *DNMT3A* and *FLT3*‐ITD mutations probably neutralized its favorable impact on survival. Based on our results showing that patients harboring concurrent *NPM1*, *FLT3*‐ITD, and *DNMT3A* mutations had the worst OS, these patients need to be scheduled for allogeneic hematopoietic stem cell transplantation in the early stage of AML. Both Metzeler and Papaemmanuil concluded that mutations in *TP53* had independent deleterious effects on survival. Besides, Papaemmanuil also confirmed the adverse effects of mutations in chromatin‐spliceosome genes and the positive effects of mutations in *IDH* ^R172^. But in our study, we did not reach the conclusions above, which might be the result of the small size of the samples.^5^, ^36^

HMAs have been widely used in clinical medicine. We confirmed their superiority in treating epigenetic‐related mutations compared to conventional chemotherapy in patients with *DNMT3A* and those with *TET2* mutations, although the difference in *TET2* mutated cohort did not reach significance. The lack of significance might be caused by the small sample size, and the relatively short follow‐up period of some patients could also be one of the reasons.

In addition, nowadays there is no specific standard of VAF cutoffs, in our study we used 1% VAF.

In summary, our study provided a detailed pattern of gene mutations and their clinical correlations and prognostic relevance in 171 Chinese AML patients based on NGS screening. We hope our work will contribute to genetic classification‐based prognostic prediction systems and improvements in patient outcomes by targeted therapeutic approaches.

## CONFLICT OF INTEREST

The authors declare no competing financial interests.

## AUTHOR CONTRIBUTION

RQW, HW, BHW and MZW had contributions in collecting clinical information of selected patients. CJC, JYQ, DWL, JW and HMZ completed the entire experimental processes. RQW and JYQ completed all statistical analyses and constructed figures. RQW wrote the manuscript. XNG and LY were the main contributors in revising the manuscript. Other authors provided some advice on the manuscript. All authors read and approved the final manuscript.

## Supporting information

Supplementary Material

Table S3

Table S5

## Data Availability

The data that support the findings of this study are available on request from the corresponding author. The data are not publicly available due to privacy.
