# Supplementary material for: Characteristics and prognostic significance of genetic mutations in acute myeloid leukemia based on a targeted next‐generation sequencing technique
Source: Cancer Med. 2020 Sep 24;9(22):8457–67. doi: 10.1002/cam4.3467 (PMC7666719; doi:10.1002/cam4.3467)
Supplement: Supplementary file 1 — Supplementary Material [file CAM4-9-8457-s001.doc]

**Supplementary Table S1 The 127 captured purpose genes according to functional classification**

| **Functional Cluster** | **Genes** |
| --- | --- |
| **Activated signaling genes** | *FLT3-ITD, NRAS, FLT3, PTPN11, KRAS, CBL, KIT, NF1, ATM, CSF3R, JAK2, NTRK1, PTEN, ETNK1* |
| **DNA methylation-associated genes** | *DNMT3A, TET2, IDH1, IDH2* |
| **Transcription factors genes** | *RUNX1, CEBPA, BCOR, GATA2* |
| **Chromatin modifier genes** | *ASXL1, EZH2, KDM6A, SETBP1, IKZF1, ETV6, STAT5B, CUX1* |
| **Nucleophosmin (NPM1) gene** | *NPM1* |
| **Tumor suppressor genes** | *TP53, WT1, PHF6* |
| **Spliceosome-complex genes** | *SRSF2, SF3B1, U2AF1, ZRSR2* |
| **Cohesin-complex genes** | *STAG2, RAD21, SMC1A, SMC3* |
| **Others** | *ABL, ELA2, PIGA, CARD11, EGFR, MEF2B, EP300, IL7R, PRPF8, TPMT, CBLB, ERCC1, MLH1, JAK1, CBLC, ERG,MTHFR, STAT5A, RB1, CCND1, FAM46C, NF2, BIRC3, JAK3, CD79B, XRCC1, GFI1, APC, PIK3CA, DDX41, DKC1, FAT1, SMAD4, NOTCH2, SYK, MDM2, BLM, FBXW7, ABCB1, CDA, GNAS, NQO1, TCF3, BRAF, ABCC3, CREBBP, GSTM1, NT5C2, TRAF3, CALR, DNAH9, GATA1, MLL, SH2B3, AKT2, CRLF2, GSTP1, MPL, CACNA1E, SRP72, SETD2, AKT3, CSF1R, HRAS, NTRK2, CDKN2A, GATA3, MYD88, AMER1, MAP3K7, CTLA4, ID3, PDGFRA, HAX1, NOTCH1, DIS3, STAT3, ATRX, CYP2C19, KMT2C, TERC, BCL2, CYP3A4, MAP2K4, TERT, BCORL1* |

**Supplementary Table S2** The list of target regions of 127 genes

| Gene | Transcript | Target Region |
| --- | --- | --- |
| *ABCB1* | NM_000927 | Exon 20, 25 |
| *ABCC3* | NM_003786 | Exon 1-2, 9, 12-13, 26-27, 29; intron 10, 13 |
| *ABL1* | NM_005157 | Exon 4-8 |
| *AKT2* | NM_001626 | Exon 2-4, 9-10, 13 |
| *AKT3* | NM_181690 | Exon 2, 5 |
| *AMER1* | NM_152424 | Exon 1 |
| *APC* | NM_000038 | Exon 1, 9-10, 15 |
| *ASXL1* | NM_015338 | Exon 12 |
| *ATM* | NM_000051 | Exon 16, 21, 43, 48, 51, 58, 62; intron 36, 40 |
| *ATRX* | NM_000489 | Exon 8-10, 17-31, 35 |
| *BCL2* | NM_000633 | full(1-3) |
| *BCOR* | NM_001123383 | full(1-14) |
| *BCORL1* | NM_021946 | full(1-13) |
| *BIRC3* | NM_001165 | Exon 2-9 |
| *BLM* | NM_000057 | Exon 9-10, 12-20, 37 |
| *BRAF* | NM_004333 | Exon 1-8, 11-13, 15-18 |
| *CACNA1E* | NM_001205293 | Exon 4, 5; intron 2 |
| *CALR* | NM_004343 | Exon 9 |
| *CARD11* | NM_032415 | Exon 4-6, 8-9 |
| *CBL* | NM_005188 | Exon 8-9, 12, 16 |
| *CBLB* | NM_170662 | Exon 8-10 |
| *CBLC* | NM_001130852 | Exon 8-10 |
| *CCND1* | NM_053056 | Exon 1, 4, 5 |
| *CD79B* | NM_000626 | Exon 5, 6 |
| *CDA* | NM_001785 | Exon 1, 2, 4 |
| *CDKN2A* | NM_000077 | full(1-4) |
| *CEBPA* | NM_004364 | Exon 1 |
| *CREBBP* | NM_004380 | Exon 25-28, 31 |
| *CRLF2* | NM_022148 | Exon 6 |
| *CSF1R* | NM_005211 | Exon 6, 21 |
| *CSF3R* | NM_000760 | Exon 12, 14-17 |
| *CTLA4* | NM_005214 | Intron 4 |
| *CUX1* | NM_181552 | full(1-24); intron 2, 16, 17, 22, 23 |
| *CYP2C19* | NM_000769 | Exon 4, 5 |
| *CYP3A4* | NM_017460 | Exon 5, 10 |
| *DDX41* | NM_016222 | Exon 5-6, 15 |
| *DIS3* | NM_014953 | Exon 10-11, 16 |
| *DKC1* | NM_001363 | Exon 1-6, 9-12, 14 |
| *DNAH9* | NM_001372 | Intron 52 |
| *DNMT3A* | NM_022552 | full(1-23) |
| *EGFR* | NM_005228 | Exon 18-19, 20-21 |
| *ELA2* | NM_001972 | Exon 2-5 |
| *EP300* | NM_001429 | full |
| *ERCC1* | NM_001983 | Exon 4 |
| *ERG* | NM_001243432 | Exon 2-10 |
| *ETNK1* | NM_018638 | Exon 3 |
| *ETV6* | NM_001987 | full(1-8) |
| *EZH2* | NM_004456 | full(1-20) |
| *FAM46C* | NM_017709 | Exon 1 |
| *FAT1* | NM_005245 | full |
| *FBXW7* | NM_033632 | Exon 8-11 |
| *FLT3* | NM_004119 | Exon 14-15, 20-21(intron14, intron15) |
| *GATA1* | NM_002049 | Exon 2 |
| *GATA2* | NM_032638 | Exon 2-6 |
| *GATA3* | NM_002051 | Exon 4 |
| *GFI1* | NM_005263 | Exon 7 |
| *GNAS* | NM_080425 | Exon 1, 8, 9 |
| *GSTM1* | NM_000561 | full(1-8) |
| *GSTP1* | NM_000852 | Exon 5 |
| *HAX1* | NM_006118 | Exon 2, 3 |
| *HRAS* | NM_005343 | Exon 1, 2 |
| *ID3* | NM_002167 | Exon 1, 2; intron 1, 2 |
| *IDH1* | NM_001282386 | Exon 2, 4 |
| *IDH2* | NM_002168 | Exon 4 |
| *IKZF1* | NM_006060 | full(1-8) |
| *IL7R* | NM_002185 | Exon 6 |
| *JAK1* | NM_002227 | Exon 9, 12 |
| *JAK2* | NM_004972 | exon10, 12, 14, 16 |
| *JAK3* | NM_000215 | exon11-13, 15-16 |
| *KDM6A* | NM_001291415 | full(1-29) |
| *KIT* | NM_000222 | Exon 2, 8-11, 13-17 |
| *KMT2A*（*MLL*） | NM_001197104 | full |
| *KMT2C* | NM_170606 | Exon 14, 43 |
| *KRAS* | NM_004985 | Exon 1, 2 |
| *MAP2K4* | NM_001281435 | Exon 9 |
| *MAP3K7* | NM_145333 | full(1-16) |
| *MDM2* | NM_002392 | Exon 3 |
| *MEF2B* | NM_001145785 | Exon 2-3, 6-8 |
| *MLH1* | NM_000249 | Exon 2-3, 13, 15, 19 |
| *MPL* | NM_005373 | Exon 10 |
| *MTHFR* | NM_005957 | Exon 4, 7 |
| *MYD88* | NM_001172566 | Exon 3-5 |
| *NF1* | NM_000267 | full |
| *NF2* | NM_000268 | Exon 8, 13 |
| *NOTCH1* | NM_017617 | Exon 26, 27, 34 |
| *NOTCH2* | NM_024408 | Exon 27, 34 |
| *NPM1* | NM_002520 | Exon 10-11, 16 |
| *NQO1* | NM_000903 | Exon 5 |
| *NRAS* | NM_002524 | Exon 1, 2 |
| *NT5C2* | NM_012229 | Exon 3-19 |
| *NTRK1* | NM_001007792 | Exon 15 |
| *NTRK2* | NM_006180 | full(1-21) |
| *PDGFRA* | NM_006206 | Exon 12, 14, 18 |
| *PHF6* | NM_001015877 | full(1-9) |
| *PIGA* | NM_002641 | full(1-7) |
| *PIK3CA* | NM_006218 | Exon 9, 20 |
| *PRPF8* | NM_006445 | Exon 28 |
| *PTEN* | NM_000314 | Exon 7, 15 |
| *PTPN11* | NM_002834 | Exon 3, 13 |
| *RAD21* | NM_006265 | full(1-14） |
| *RB1* | NM_000321 | full(1-27) |
| *RUNX1* | NM_001754 | full(1-9) |
| *SETBP1* | NM_015559 | Exon 3, 4 |
| *SETD2* | NM_014159 | Exon 2-5, 8, 12, 14-16, 19-21 |
| *SF3B1* | NM_012433 | Exon 13-16 |
| *SH2B3* | NM_005475 | Exon 1-3, 6 |
| *SMAD4* | NM_005359 | Exon 2,10 |
| *SMC1A* | NM_006306 | Exon 2-3, 11, 13-14, 16-17 |
| *SMC3* | NM_005445 | Exon 9-10, 13, 19, 23, 25, 28 |
| *SRP72* | NM_006947 | Exon 6, 7 |
| *SRSF2* | NM_003016 | Exon 1, 2 |
| *STAG2* | NM_001042749 | full(1-35) |
| *STAT3* | NM_003150 | Exon 21 |
| *STAT5A* | NM_003152 | Exon 15-16, 19, 20 |
| *STAT5B* | NM_012448 | Exon 14-16 |
| *SYK* | NM_001135052 | Exon 5 |
| *TCF3* | NM_003200 | Exon 17 |
| *TERC* | NR_001566 | full |
| *TERT* | NM_198253 | Exon 1-2, 4-12, 14-16 |
| *TET2* | NM_001127208 | Exon 1-11 |
| *TP53* | NM_000546 | Exon 2-11 |
| *TPMT* | NM_000367 | Exon 3, 5, 8 |
| *TRAF3* | NM_145725 | Exon 1, 3-8, 10 |
| *U2AF1* | NM_006758 | Exon 2, 6 |
| *WT1* | NM_024426 | Exon 3, 6-9 |
| *XRCC1* | NM_006297 | Exon 10 |
| *ZRSR2* | NM_005089 | full(1-11) |

**Supplementary Table S3** The sequencing metrics of 171 AML samples (Specificity = On target reads before duplication removal / Total mapped reads before duplication removal; Clean data means uniquely mapped data (Filter“Not Primary Alignment”); The minimum cutoff for read depth in the“Coverage” column is 1x which mainly used for the verification of abnormalities during probe capture process. The minimum cutoff of read depth used for variant calling is 30x).

**Supplementary Table S4** The detail information of cytogenetics and all variants detected in 181 patients.

| Patients ID | Cytogenetics | Genetic mutations |
| --- | --- | --- |
| HB15BA00146 | Undetermined | *TET2*: c.3779A>G p.N1260S  *FLT3*: c.2504A>C p.D835A  *FLT3*: c.1775T>A p.V592D  *STAG2*: c.500_503del p.Q167fs |
| HB15BA00253 | 46, XY | *IDH2*: c.419G>A p.R140Q  *ASXL1*: c.1888_1910del p.H630fs  *CEBPA*: c.737dupT p.L246fs  *CEBPA*: c.275dupA p.K92fs  *SRSF2*: c.284C>A p.P95H  *STAG2*: c.992dupA p.Y331_V332delinsX |
| HB15AN00163 | Complex karyotype | *DNMT3A*: c.1740delC p.P580fs  *NRAS*: c.38G>A p.G13D  *NRAS*: c.35G>A p.G12D  *TP53*: c.713G>A p.C238Y |
| HB15BA00120 | t(8,21) | *KRAS*: c.35G>A p.G12D |
| HB15AN00103 | 46, XY |  |
| HB15BA00178 | 46, XY | *NPM1*: c.859_860insTCTG p.L287fs  *NRAS*: c.35G>A p.G12D  *TET2*: c.1261G>T p.E421X  *TET2*: c.2512dupA p.N837fs  *RUNX1*: c.1438_1633del p.Y480fs  *SRSF2*: c.284C>T p.P95L  *RAD21*: c.1126-1G>A |
| HB15BA00147 | 46, XY | *NPM1*: c.859_860insTCTG p.L287fs  *BCOR*: c.4326+1G>A |
| HB15AN00170 | Undetermined | *TP53*: c.578A>G p.H193R |
| HB15BA00204 | 46, XY | *CSF3R*: c.2346dupC p.S783fs  CBL:c.1096_1106delinsATTCCCCC  p.E366_E369delinsIPP |
| HB15AN00165 | del(12p) | *RUNX1*: c.299C>T p.S100F  *RUNX1*: c.619C>T p.R207W  *WT1*: c.1128_1129insGGCG p.T377fs  *CBL*: c.T1139T>C p.L380P |
| HB15BA00200 | t(8,21) | *TP53*: c.466C>T p.R156C  *FLT3*: c.1775T>C p.V592A  *SRSF2*: c.284C>T p.P95L  *CSF3R*: c.2346dupC p.S783fs |
| HB15AN00142 | Undetermined | *TET2*: c.4633C>T p.Q1545X  *TET2*: c.3589A>G p.K1197E  *ASXL1*: c.2324T>G p.L775X  *SRSF2*: c.3894dupT p.C1298fs |
| HB15AN00214 | 46, XX | *CEBPA*: c.166dupT p.C56fs  *CEBPA*: c.912_913insTTG p.Q305delinsLQ |
| HB15AN00174 | 46, XX | *JAK2*: c.1849G>T p.V617F |
| HB15BA00119 | 46, XY | *DNMT3A*: c.2644C>T p.R882C  *NPM1*: c.859_860insTCTG p.L287fs  *IDH2*: c.419G>A p.R140Q  *RUNX1*: c.611G>A p.R204Q  *FLT3*: c.2505T>G p.D835E  *SRSF2*: c.284C>T p.P95L |
| HB15BA00174 | 46, XY | *NRAS*: c.181C>A p.Q61K  *IDH2*: c.419G>A p.R140Q  *WT1*: c.871_872+2delinsTA  *BCOR*: c.4879C>T p.R1627X |
| HB15AN00239 | 46, XY | *DNMT3A*: c.2645G>A p.R882H  *NPM1*: c.859_860insTCTG p.L287fs  *NRAS*: c.35G>A p.G12D  *NRAS*: c.34G>A p.G12S  *CEBPA*: c.68dupC p.P23fs  *ZRSR2*: c.425delA p.Q142fs |
| HB15BA00261 | t(8,21) | *RUNX1*: c.934dupA p.T312fs  *U2AF1*: c.101C>A p.S34Y  *IDH1*: c.394C>T p.R132C |
| HB15AN00266 | 46, XX | *DNMT3A*: c.2645G>A p.R882H  *NPM1*: c.859_860insTCTG p.L287fs |
| HB15AN00068 | Complex karyotype | *SF3B1*: c.2098A>G p.K700E |
| HB15AN00270 | Undetermined | *PTEN*: c.701_702insC p.R234fs |
| HB15AN00178 | 46, XY | *TET2*: c.1545dupC p.N515fs  *CEBPA*: c.68_69insCC p.P23fs  *CEBPA*: c.937_939del p.313_313del  *JAK2*: c.1849G>T p.V617F |
| HB15AN00180 | 46, XY | *U2AF1*: c.470A>C p.Q157P  *IDH1*: c.394C>T p.R132C |
| HB15AN00213 | 46, XY | *CEBPA*: c.68delC p.P23fs  *CEBPA*: c.914A>C p.Q305P  *CSF3R*: c.1919C>A p.T640N |
| HB15BA00157 | 46, XY | *CEBPA*: c.936_937insCAG p.K313delinsQK  *CEBPA*: c.293dupC p.T98fs |
| HB15AN00219 | 46. XX | *CEBPA*: c.92_99del p.F31fs  *CEBPA*: c.936_937insCAG p.K313delinsQK |
| HB15AN00160 | 46. XX | *NRAS*: c.38G>T p.G13V  *CEBPA*: c.289_290insGGCC p.P97fs  *CEBPA*: c.937_939del p.313_313del  *GATA2*: c.952G>A p.A318T |
| HB15BA00171 | +8  t(7,11)(p15,p15) | *TET2*: c.2113C>T p.Q705X  *IDH2*: c.418C>T p.R140W  *U2AF1*: c.467G>A p.R156H  *EZH2*: c.1408C>T p.Q470X  *EZH2*: c.1978G>A p.G660R |
| HB15BA00151 | 46, XY | *DNMT3A*: c.2644C>T p.R882C  *NPM1*: c.859_860insTCTG p.L287fs  *FLT3*: c.1352C>T p.S451F |
| HB15AN00227 | 46, XY | *NPM1*: c.859_860insTCTG p.L287fs  *IDH1*: c.394C>A p.R132S |
| HB15BA00167 | Complex karyotype |  |
| HB15BA00158 | Complex karyotype | *DNMT3A*: c.2645G>A p.R882H  *NRAS*: c.38G>A p.G13D  *NRAS*: c.37G>C p.G13R |
| HB15BA00262 | 46, XY | *DNMT3A*: c.2645G>A p.R882H  *NPM1*: c.859_860insTCTG p.L287fs  *CALR*: c.1102_1104del p.368_368del |
| HB15AN00264 | IR-AML | *DNMT3A*: c.2683G>A p.V895M  *NPM1*: c.861_862insTGCA p.L287fs  *TET2*: c.4120T>A p.C1374S  *FLT3*: c.2503G>T p.D835Y  *WT1*: c.1144_1145insTCGG p.A382fs |
| HB15AN00184 | 46, XX | *DNMT3A*: c.2645G>A p.R882H  *NPM1*: c.859_860insTCTG p.L287fs  *PTPN11*: c.213T>G p.F71L |
| HB15AN00143 | 46, XX | *DNMT3A*: c. 2645G>A p.R882H  *NPM1*: c.859_860insTCTG p.L287fs  *TET2*: c.2538_2541del p.E846fs |
| HB15AN00265 | 46, XX | *NPM1*: c.865_866insTGAA p.Q289fs  *IDH2*: c.419G>A p.R140Q  *CEBPA*: c.927dupG p.T310fs  *CEBPA*: c.539delC p.P180fs  *SRSF2*: c.170T>A p.F57Y |
| HB15AN00205 | +8  +4 | *NRAS*: c.35G>A p.G12D  *IDH2*: c.515G>A p.R172K  *BCOR*: c.4195C>T p.Q1399X |
| HB15BA00471 | IR-AML | *NRAS*: c.38G>A p.G13D  *WT1*: c.1142C>A p.S381X  *CEBPA*: c.530dupG p.G177fs  *IDH1*: c.395G>A p.R132H  *CSF3R*: c.1853C>T p.T618I |
| HB15AN00231 | 46, XX | *NRAS*: c.35G>A p.G12D  *KRAS*: c.35G>T p.G12V  *GATA2*: c.1140C>A p.H380Q  *GATA2*: c.1082G>A p.R361H |
| HB15AN00238 | Complex karyotype | *ASXL1*: c.1773C>A p.Y591X  *RUNX1*: c.485G>A p.R162K  *SRSF2*: c.284C>A p.P95H  *STAT5B*: c.1883C>G p.T628S |
| HB15AN00255 | 46,XX | *DNMT3A*: c.2645G>A p.R882H  *NPM1*: c.859_860insTCTG p.L287fs  *U2AF1*: c.101C>T p.S34F  *FLT3*: c.2503G>T p.D835Y  *PTPN11*: c.1504T>C p.S502P |
| HB15AN00247 | t(8,21) | *KIT*: c.2466T>G p.N822K  *KIT*: c.2464A>T p.N822Y  *RAD21*: c.481+2->GGTTG |
| HB15AN00249 | 46, XX | *DNMT3A*: c.2578T>C p.W860R  *NPM1*: c.859_860insTCTG p.L287fs  *FLT3*: c.2516A>G p.D839G  *CBL*: c.1259G>A p.R420Q |
| HB15BA00451 | 46, XX | *TET2*: c.5085delT p.G1695fs  *TET2*: c.3961delA p.K1321fs |
| HB15BA00559 | 46, XX | *DNMT3A*: c.2644C>T p.R882C  *NPM1*: c.859_860insTCTG p.L287fs  *NRAS*: c.38G>A p.G13D  *IDH2*: c.419G>A p.R140Q |
| HB15BA00617 | t(8,21) | *NRAS*: c.38G>A p.G13D  *GATA2*: c.818dupG p.G273fs |
| HB15BA00423 | Complex karyotype | *SMC1A*: c.1756C>T p.R586W |
| HB15AN00241 | 46, XY | *NPM1*: c.859_860insTCTG p.L287fs  *PTPN11*: c.1504T>G p.S502A  *CYP3A4*: c.878T>C p.L293P  *ETV6*: c.744_745del p.S248fs  *RAD21*: c.1756C>T p.R586X |
| HB15BA00117 | t(8,21)  -Y |  |
| HB15BA00112 | +8 | *NPM1*: c.869_875delGGAGGAAinsCCTTCTCCCTC p.W290fs |
| HB15BA00621 | 46, XX | *DNMT3A*: c.2645G>A p.R882H  *NPM1*: c.859_860insTCTG p.L287fs  *NRAS*: c.35G>A p.G12D  *TET2*: c.4182+1G>C  *TET2*: c.2123C>G p.S708X  *PTPN11*: c.1520C>A p.T507K  *PTPN11*: c.1471C>T p.P491S |
| HB15BA00598 | 46, XY | *ASXL1*: c.3671G>C p.R1224T  *ASXL1*: c.3671G>C p.R1224T  *SRSF2*: c.284C>A p.P95H |
| HB15AN00243 | 46, XX | *NPM1*: c.859_860insTCTG p.L287fs  *IDH2*: c.419G>T p.R140L |
| YB15AN00007 | 46, XX |  |
| HB15AN00001 | t(11;20)(p15;q11)  +mar | *DNMT3A*: c.1668-2A>C  *WT1*: c.1109_1110insC p.R370fs  *WT1*: c.1107A>G p.R369R |
| HB15AN00006 | t(8,21) | *KIT*: c.2466T>G p.N822K  *KIT*:  c.1248_1257GACTTACGAC>ATCC; p.Leu416fs |
| HB15AN00008 | 46, XY | *ASXL1*: c.1888_1910del23 p.H630fs |
| HB15AN00011 | t(10;11)(p13;q13) |  |
| HB15AN00022 | t(8,21) |  |
| HB15AN00020 | Complex karyotype | *DNMT3A*: c.2121_2122del p.G707fs  *DNMT3A*: c.1608C>A p.Y536X  *FLT3*: c.2503G>T p.D835Y  *BCOR*: c.2707_2708insGAGC; p.P903fs  *IDH1*: c.394C>T p.R132C |
| HB15AN00016 | 46, XX |  |
| HB15AN00037 | 46, XX |  |
| HB15AN00030 | t(7;11)(p15;p15) | *NRAS*: c.G38A p.G13D  *WT1*: NA |
| HB15AN00034 | t(8,21)  del(12p) |  |
| HB15AN00042 | t(9,22) | *NPM1*: c.859_860insTCTG p.L287fs |
| HB15AN00054 | +11 | *IDH2*: c.515G>A p.R172K |
| HB15AN00062 | t(8,21) |  |
| HB15AN00058 | +9 | *TET2*: c.G3451T p.E1151X |
| HB15AN00049 | Complex karyotype | *TP53*: c.G524A p.R175H |
| HB15AN00081 | 46, XY |  |
| HB15AN00067 | Add(10)(p15) | *NRAS*: c.35G>C p.G12A |
| HB15AN00085 | t(6;9)  del(8)(q21) |  |
| HB15AN00086 | 46, XY | *NRAS*: c.38G>T p.G13V  *WT1*: c.1144_1145insTCGG p.A382fs |
| HB15AN00093 | 46, XY | *DNMT3A*: c.2645G>A p.R882H  *KRAS*: c.35G>T p.G12V |
| HB15AN00098 | Undetermined | *WT1*: c.1140_1141insCTCTTGTACGG p.S381fs |
| D132071 | 46, XX | *HOXA9*: c.377C>G p.S126C  *KMT2B*: c.5741G>A p.R1914H  *NRAS*: c.34G>T p.G12C  *SF3B2*: c.512C>T p.S171L  *WT1*: c.1237C>T p.R413X |
| D132072 | Complex karyotype | *HOXD11*: c.734G>A p.G245D |
| D132073 | 46, XY | *CEBPA*: c.936_937insCAG p.K313delinsQK  *CEBPA*: c.175G>T        p.E59X  *NOTCH3*: c.6334G>A p.G2112S  *RAD21*: c.1470+2T>C |
| D132074 | Complex karyotype | *TP53*: c.428G>A p.C143Y |
| D132076 | 9q-  hypodiploid | *CEBPA*: c.68_78del        p.P23fs  *WT1*: c.1089dupG p.S364fs |
| D132077 | +22 | *ASXL1*: c.1379G>A        p.G460E  *NRAS*: c.35G>A p.G12D  *RNF213*: c.2852A>G p.H951R  *TP53*: c.140A>G p.H47R  *WHSC1*: c.304T>A p.S102T |
| D132081 | 46, XY | *DNMT3A*: c.2645G>A p.R882H  *NPM1*: c.772_773insTCTG p.L258fs  *TET2*: c. 2224C>T p. Q742X  *FLT3*-ITD: insert36bp |
| D132083 | t(1;8)(p35;q12)  hypodiploid | *CEBPA*: c.951_952insCTG p.T318delinsLT  *CEBPA*: c.247delC        p.Q83fs  *DNMT3A*: c.1920_1923del p.F640fs  *NUTM1*: c.442G>A p.V148I  *WT1*: c.1172T>A p.L391X  *WT1*: c. 1089delG p. R363fs |
| D132084 | 46, XX | *ASXL1*: c.1927delG        p.G643fs  *SMC3*: c.121dupT p.N40fs |
| D132085 | 46, XY | *CEBPA*: c.939_940insAAG p.V314delinsKV  *CEBPA*: c. 259C>T        p.Q87X  *FIP1L1*: c.34G>C p.E12Q  *NRAS*: c.182A>G p.Q61R  *NRG3*: c.418C>A p.P140T  *TAF15*: c.1443_1444ins p.G481delins  *TP53*: c.473G>T p.R158L  *TPM3*: c.124C>T p.R42W  *WT1*: c.1089dupG p.S364fs |
| D132086 | 46, XY | *NRAS*: c.182A>G p.Q61R  *TLX3*: c.706G>A p.A236T |
| D132087 | t(9;22) | *FLT3*: c.2503G>T        p.D835Y  *NPM1*: c.772_773insTCTG p.L258fs  *WT1*: c.1250G>C p.R417P |
| D132821 | Undetermined | *AXCL1*: c.150_164del p.50_55del  *CEBPA*: c.914_915insAGGA        p.Q305fs  *CEBPA*: c.912_913insGC p.Q305fs  *CEBPA*: c.332_339del p.A111fs  *PBX1*: c.519C>G p.N173K |
| D132822 | 46, XY | *C19orf10*: c.182A>G p.Y61C |
| D132825 | 46, XX | *NPM1*: c.772_773insTCTG p.L258fs  *FLT3*-ITD: insert24bp |
| D132827 | 46, XY | *KRAS*: c.38G>A         p.G13D |
| D132828 | Undetermined | *FOXP1*: c.159C>A p.H53Q  *GOLGA4*: c.1046G>A p.R349H  *NUTM1*: c.1370A>G p.Q457R |
| D132830 | 46, XX | *CEBPA*: c.930_931insACG p.Q311delinsTQ  *CEBPA*: c.247_256del        p.Q83fs  *LONP1*: c.1738A>G p.T580A |
| D132832 | 9q- | *CEBPA*: c.939_940insAAG p.V314delinsKV  *DOT1L*: c.3050A>G        p.Q1017R  *WT1*: c.1086_1096del p.V362fs  *FLT3*-ITD: insert66bp |
| D132838 | 46, XX | *ATM*: c.274A>G p.K92E  *CEBPA*: c.196_197insT        p.A66fs  *RPN1*: c.1696G>A p.A566T  *TLR9*: c.1801C>T p.R601W |
| D132839 | t(16;21)(p11;q22) | *KMT2B*: c.5501G>A p.R1834H |
| D132841 | Complex karyotype | *ASXL1*: c.2176A>T        p.K726X  *NRAS*: c.182A>G p.Q61R  *RAD50*: c.2047G>A p.V683I  *SRSF2*: c.284C>A p.P95H  *STAG2*: c.3130_3131insG p.Y1044_R45delinsX |
| D132842 | 46, XX | *DNMT3A*: c.2645G>A p.R882H  *IDH2*: c.419G>A p.R140Q  *NPM1*: c.772_773insTCTG p.L258fs  *NR4A3*: c.508G>C p.G170R |
| D132843 | 46, XX | *BIRC5*: c.383G>A        p.S128N  *IDH1*: c.394C>G p.R132G  *ITGA2*: c.89A>C p.Y30S  *NPM1*: c.772_773insTCTG p.L258fs  *FLT3*-ITD: insert60bp |
| D132844 | 46, XX | *GOLGA4*: c.3277G>C p.E1093Q  *TP53*: c.100G>A p.V34M  *WT1*: c.1094_1095ins p.A365fs |
| D132846 | 46, XY | *CEBPA*: c.324 C>A        p.Y108X  *GAS7*: c.223G>A p.G75S  *TET2*: c.4659_4674del p.Q1553fs |
| D132848 | 46, XX | *FANCA*: c.356C>G        p.S119C  *IDH2*: c.419G>A p.R140Q  *NPM1*: c.772_773insTCTG p.L258fs |
| D132849 | 46, XY | *MBD1*: c.586C>T p.R196C  *WHSC1L1*: c.2031T>G p.D677E |
| D132850 | +11 | *ATM*: c.146C>G p.S49C  *KMT2B*: c.7111G>A p.D2371N  *RUNX1*: c.1210dupC p.H404fs  *U2AF1*: c.101C>T p.S34F |
| D132851 | 9q- | *CEBPA*: c.941_946del p.314_316del  *CEBPA*: c.68dupC        p.P23fs  *NFE2*: c. 661dupG p.E221fs  *PSMD2*: c.526G>A p.A176T  *STAG2*: c.2887C>T p.Q963X |
| D132853 | tas(19;21)(q13;q22)  hypodiploid | *CCDC88C*: c.3803T>A p.L1268Q  *KRAS*: c.38G>A         p.G13D  *NOTCH3*: c. 1715C>T p.P572L  *RUNX1T1*: c.92G>A p.R31H  *SPRY4*: c. 643A>G p.T215A |
| D132854 | 46, XX | *ESR1*: c.1026G>A        p.M342I  *FNBP1*: c.1406G>A         p.R469Q  *NPM1*: c.772_773insTCTG p.L258fs  *PTPN11*: c.227A>T p.E76V |
| D132855 | Complex karyotype | *DNMT3A*: NA  *DNMT3A*: c.1916delT p.L639fs  *RAD50*: c.3931G>A p.V1311I  *STAG2*: c.1570_71insTAAT p.I524fs  *TET2*: c.3643G>T p.E1215X  *U2AF1*: c.101C>T p.S34F |
| D132856 | 46, XY | *DNMT3A*: c.2645G>A p.R882H  *NPM1*: c.772_773insTCTG p.L258fs  *PTPN11*: c.215C>T p.A72V |
| D132857 | 46, XY | *DNMT3A*: c.2644C>T p.R882C  *DOT1L*: c.4229C>G        p.A1410G  *IDH1*: c.395G>A p.R132H  *MBD1*: c.853G>A p.V285M  *NPM1*: c.772_773insTCTG p.L258fs |
| D132858 | 46, XY | *CEBPA*: c.939_940insAAG p.V314delinsKV |
| D132859 | 46, XY | *NRAS*: c.37G>C p.G13R |
| D132860 | Complex karyotype | *ABCA2*: c.275C>G        p.T92R  *FLT3*: c.1988A>T p.K663M  *RAD21*: c.54G>T p.W18C |
| D132861 | 46, XY | *ASXL1*: c. 1927dupG p.G642fs  *DNMT3A*: c.2645G>A p.R882H  *IDH2*: c.419G>A p.R140Q  *MLLT11*: c.133G>A p.V45I  *NOTCH3*: c.6665C>T p.P2222L  *SRSF2*: c.284C>A p.P95H |
| D132862 | 46, XX | *BRINP3*: c.383G>A        p.S128N  *FNBP1*: c. 301G>T        p.D101Y  *IDH2*: c.419G>A p.R140Q  *NPM1*: c.772_773insTCTG p.L258fs  *TP53*: c.214C>G p.L72V |
| D132863 | 46, XX | *CEBPA*: c.919_921linsAGGC p.N307dellinsKGH  *CEBPA*: c.196_197insTAGG        p.A66fs  *IDH2*: c.472G>A p.G158S  *MSH4*: c.2401G>A p.D801N  *NUTM1*: c.628G>A p.V210I |
| D132865 | Complex karyotype | *DOT1L*: c.3023C>T p.S1008F  *RUNX1*: c.1415T>C p.L472P  *TET2*: c.3730_3731del p. L1244fs |
| D132953 | 46, XY | *CCDC88C*: c.6026C>T        p.P2009L  *CEBPA*: c.890G>C        p.R297P  *HOXD13*: c.168_179del p.56_60del |
| D132956 | 46, XX | *PRAME*: c.1514G>A p.C505Y  *TET2*: c.822delC p.I274fs  *TET2*: c.3015delG p.K1005fs |
| D132957 | 46, XY | *ASXL1*: c. 1927dupG p.G642fs  *IDH2*: c.515G>A p.R172K  *RAD21*: c.1388_1389del p.463_463del |
| D132959 | 46, XX | *DNMT3A*: c.2644C>T p.R882C  *EVI2A*: c.104T>C p.L35P  *NPM1*: c.772_773insTCTG p.L258fs  *FLT3*-ITD: insert66bp |
| D132960 | Undetermined | *FLT3*: c.229G>A p.E77K  *NRAS*: c.181C>A p.Q61K  *U2AF1*: c.101C>A p.S34Y |
| D132961 | 46, XY | *ASXL1*: c.1888_1910del        p.H630fs  *CBLB*: c.2936G>A        p.R979H  *DNMT3A*: c.2645G>A p.R882H  *IDH2*: c.419G>A p.R140Q  *MBD1*: c.1430G>C p.S477T  *RUNX1*: c.1132dupC p.H378fs  *U2AF1*: c.470A>C p.Q157P |
| D132963 | Complex karyotype | *ASXL1*: c.1643_1644ins        p.F548fs  *RUNX1*: c.1189dupC p.Q397fs  *TET2*: c.601delA p.K201fs |
| D132964 | 46, XY | *ABCC9*: NA  *DNMT3A*: c.2200T>A p.F734I  *NPM1*: c.772_773insTCTG p.L258fs  *PTPN11*: c.1508G>C p.G503A |
| D132965 | 46, XX | *IDH1*: c.394C>T p.R132C  *SRSF2*: c.284C>A p.P95H  *STAG2*: c.2185-2A>T |
| D132966 | +11 | *ASXL1*: c.4243C>T        p.R1415X  *ATM*: c.2537T>C p.L846P  *ETV6*: c.346delC p.L116fs  *ETV6*: c.348_352del p.L116fs  *FBXW7*: c.535C>T        p.R179C  *LONP1*: c.2035C>T p.R679C  *U2AF1*: c.101C>T p.S34F |
| D132967 | 46, XY | *CEBPA*: c.929_930insTCT         p.T310delinsTL  *NRAS*: c.38G>A p.G13D  *TLX1*: c.355A>G p.S119G  *WT1*: c.1321C>T p.R441X |
| D132968 | +8  t(3;12)(q24;p13) | *DNMT3A*: c.2645G>A p.R882H  *IDH2*: c.419G>A p.R140Q  *KDM6A*: c.619+1G>A  *WT1*: c.1289G>T p.G430V  *FLT3*-ITD: insert21bp |
| D132969 | -20 | *CEBPA*: c.878_880del        p.293_294del  *ID4*: c.379C>G p.P127A  *JAK2*: c.1849G>T p.V617F  *KMT2B*: c.1885C>T p.P629S |
| D132970 | 46, XX | *CEBPA*: c.939_940insAAG p.V314delinsKV  *CEBPA*: c.68dupC        p.P23fs  *EHMT1*: c. 275C>T        p.A92V |
| D132971 | 46, XY | *CEBPA*: c.209delC        p.P70fs  *KIT*: c.2447A>T p.D816V |
| D132974 | 46, XY | *BTG1*: c.116C>A p.T39N  *NFE2*: c.661dupG p.E221fs |
| D132975 | 46, XY | *CEBPA*: c.939_940insAAG p.V314delinsKV  *CEBPA*: c.107delG        p.G36fs  *FLT3*-ITD: insert21bp |
| D132976 | 46, XY | *BAX*: c.118C>T        p.R40X  *ITGA2*: c.2269C>T p.R757C  *NF1*: c.6802G>A p.V2268I  *NPM1*: c.772_773insTCTG p.L258fs  *PICALM*: c.64G>C p.V22L |
| D132977 | 46, XY | *CD28*: c.236G>A        p.R79H  *GOLGA4*: c.2192A>T p.H731L  *NRAS*: c.182A>G p.Q61R |
| D132978 | 46, XX | *ABCC9*: c.2530A>G        p.I844V  *CD28*: c.298C>T p.R100C  *DNMT3A*: c.2645G>C p.R882P  *LYL1*: c.436C>A p.P146T  *NPM1*: c.772_773insTCTG p.L258fs  *PAX7*: c.434G>A p.R145Q  *FLT3*-ITD: insert21bp |
| D132979 | 9q- | *CEBPA*: c.945_946insCTG p.E316delinsLE  *RABEP1*: c.2054G>A p.R685H |
| D132980 | 46, XX |  |
| D132981 | 46, XX | *CEBPA*: c.286_296del        p.G96fs  *IDH2*: c.419G>A p.R140Q  *NCKIPSD*: c.487_490del p.I163fs  *RUNX1*: c.1190A>G p.Q397R  *SMC3*: c.430-1G>A |
| D132982 | 46, XY | *FANCA*: c.2291G>A         p.R764Q  *KRAS*: c.38G>A         p.G13D  *MYC*: c.992G>C p.R331P  *SF3B1*: c.2111T>C p.I704T  *U2AF1*: c.101C>T p.S34F |
| D132983 | 46, XX | *CEBPA*: c.937_939del p.313_313del  *CEBPA*: c.196delG        p.A66fs  *KIT*: c.2446G>T p.D816Y  *TET2*: c.727C>T p.Q243X |
| D132984 | t(11;17) |  |
| D132985 | 46, XX | *DNMT3A*: c.2645G>A p.R882H  *NPM1*: c.772_773insTCTG p.L258fs  *TET2*: c.3578G>A p.C1193Y  *FLT3*-ITD: insert36bp |
| D132986 | Undetermined | *CEBPA*: c.939_940insAAG p.V314delinsKV  *CEBPA*: c.247delC        p.Q83fs  *MMACHC*: c.641G>A p.R214H |
| D132988 | 46, XX | *CXCR4*: c. 685T>A        p.S229T  *HOXD11*: c.734G>A p.G245D  *KMT2B*: c.1378G>C p.V460L  *NRAS*: c.35G>A p.G12D |
| D132989 | +13  +22 | *CBLB*: c.2556T>G        p.F852L  *DNMT3B*: c.1596T>G p.C532W  *IDH2*: c.419G>A p.R140Q  *RNF213*: c.11812G>A p.E3938K  *STRN*: c.581C>T p.T194M  *U2AF1*: c.104G>A p.R35Q |
| D132990 | 46, XX | *CEBPA*: c.936_937insCAG p.K313delinsQK  *CEBPA*: c.232delC        p.L78fs  *FANCA*: c.3532G>A         p.E1178K  *LASP1*: c.298G>A p.V100I |
| D132991 | 46, XX | *NTRK1*: c.652G>A p.E218K  *PHF6*: c.751C>T p.Q251X  *TTL*: c.904A>G p.I302V |
| D132993 | +8 |  |
| D132994 | Undetermined | *NPM1*: c.772_773insTCTG p.L258fs  *TET2*: c.4132T>C p.C1378R |
| D132995 | 46, XY | *KMT2B*: c.6488T>A p.F2163Y  *SPRY4*: c.745T>C p.C249R  *FLT3*-ITD: insert78bp |
| D132996 | 46, XY | *CEBPA*: c.542dupA        p.Y181_Q182delinsX  *DOT1L*: c.1455G>C        p.Q485H  *IDH2*: c.419G>A p.R140Q |
| D132997 | Undetermined | *ABCG8*: c.211T>A        p.F71I  *NPM1*: c.772_773insTCTG p.L258fs |
| D132999 | +8 | *ATM*: c.283C>A p.Q95K  *PTPN11*: c.179G>T p.G60V |
| D133000 | 9q- | *STIL*: c. 227C>T p. S76L  *WT1*: NA  *FLT3*-ITD: insert21bp |
| D133002 | 46, XY |  |
| D133006 | 46, XY | *CEBPA*: c.936_937insCAG p.K313delinsQK  *CEBPA*: c.183delC        p.S61fs  *MAF*: c.1178A>C p.Q393P  *MYC*: c.221C>G p.P74R |
| D133128 | Complex karyotype | *DNMT3A*: c.2645G>A p.R882H  *MSH4*: c.397A>C p.K133Q  *NPM1*: c.772_773insTCTG p.L258fs  *FLT3*-ITD: insert63bp |
| D133118 | 46, XX | *DNMT3B*: c.1804G>A p.V602I |
| D133120 | hypodiploid  +mar | *CEBPA*: c.919_921del p.307_307del  *CEBPA*: c.68dupC        p.P23fs  *TAF15*: c.1372_1395del p.458_465del |
| D132954 | Undetermined | *CCDC88C*: c.1878G>C p.K626N  *IDH2*: c.419G>A p.R140Q  *NPM1*: c.772_773insTCTG p.L258fs  *NTRK1*: c.295G>A p.V99M  *RUNX1T1*: c.946G>A p.E316K  *SRSF2*: c.284C>A p.P95H |
| D132962 | t(16; 21)(p11; q22) | *DNMT1*: c.879_880ins        p.A294_A295delins  *DNMT1*: c.445_446ins        p.P149_E150delins  *STAG2*: c.796_798del p.266_266del |
| D133007 | 46, XY | *AXCL1*: c.149_150ins p.A50delins  *FLT3*: c.1739A>C p.Q580P  *FOXO4*: c. 902A>G         p.N301S |
| D133009 | 46, XX | *DNMT3A*: c.2162C>T        A721V  *NPM1*: c.774_775insTGCA p.L258fs  *FLT3*-ITD: insert51bp |
| D132864 | Complex karyotype | *CD44*: c.968C>G        p.P323R  *FGFR3*: c.1211A>G        p.K404R |
| HB15BA00660 | +8 | *STAG2*: c.1970dupT p.L657fs |
| HB15BA00649 | 9q- | *WT1*: c.1109_1110insGT p.R370fs  *CEBPA*: c.939_940insTCT p.V314delinsSV  *CEBPA*: c.69delG p.P23fs  *EZH2*: c.1942G>A p.G648R  *CSF3R*: c.1853C>T p.T618I |
| HB15BA00697 | 46, XX | *DNMT3A*: c.2645G>A p.R882H  *NPM1*: c.859_860insTCTG p.L287fs  *FLT3*: c.2504A>C p.D835A  *FLT3*: c.2503G>T p.D835Y  *KRAS*: c.35G>T p.G12V  *PTPN11*: c.218C>T p.T73I  *PTPN11*: c.1504T>G p.S502A  *SMC3*: c.1142G>A p.R381Q |
| HB15BA00571 | t(8;21) | *KIT*: c.2447A>T p.D816V |
| HB15BA00669 | 46, XX | *WT1*: c.1144_1145insTCGG p.A382fs |
| HB15BA00818 | 46, XX | *NPM1*: c.861_862insTGCA p.L287fs  *FLT3*: c.1780T>G p.F594V  *FLT3*: c.1471G>T p.V491L |
| HB15BA00674 | Undetermined | *RUNX1*: c.1262_1275del p.G421fs |
| HB15BA00643 | Undetermined | *PTPN11*: c.226G>C p.E76Q  *KIT*: c.1253_1256delinsTC p.Y418_D419delinsF  *KIT*: c.1248_1250del p.416_417del  *KIT*: c.1526A>T p.K509I |
| HB15BA00762 | 46, XX | *TET2*: c.4738delC p.P1580fs  *PTPN11*: c.1530G>T p.Q510H |
| HB15JXBA00063 | Undetermined | *NPM1*: c.859_860insTCTG p.L287fs  *RAD21*: c.971_972delinsATTGGATCTGATTGCCTTATTTAAGTAYGA p.L324delinsHWIX |
| HB15JXBA00064 | Undetermined | *KRAS*: c.182A>T p.Q61L |
| HB15JXBA00050 | Undetermined | *U2AF1*: c.101C>A p.S34Y  *BCOR*: c.3547C>T p.R1183X  *SF3B1*: c.2219G>A p.G740E  *SETBP1*: c.2602G>A p.D868N  *IKZF1*: c.1000delC p.P334fs  *NF1*: c.888+2T>G  *ETNK1*: c.731A>G p.N244S |
| HB15JXBA00062 | Undetermined |  |
| HB15JXBA00051 | Undetermined | *WT1*: c.1138_1140delinsACGGG p.R380fs  *CEBPA*: c.945_946insAAGCAGCGCAACGTGGAGACGCAGCAGAAGGTGCTG p.E316delinsKQRNVETQQKVLE  *CEBPA*: c.69dupG p.H24fs |

**Supplementary Table S5** The VAF information of 108 samples of patients from 2015 to 2017.

**Supplementary Table S6** Association analysis between genetic mutation and clinical characteristics in newly diagnosed AML patients

| **Mutation** | | **Age**  **median^a^, range** | **Gender**  **male/female^b^** | **Cytogenetics**  **low/intermediate/high risk^c^** | **Hemoglobin**  **median^a^, g/L** | **WBC**  **median^a^,×10^9^/L** | **Platelets**  **median^a^, ×10^9^/L** | **BM Blast**  **median^a^, %** | **FAB subtype**  **M1/M2/M4/M5/M6^c^** |
| --- | --- | --- | --- | --- | --- | --- | --- | --- | --- |
| ***FLT3*** | **ITD** | 48(20-73) | 9/22 | 0/23/4 | 80(36-122) | 33.8(3-333) | 37(5-200) | 66(21-94) | 1/5/15/7/1 |
|  | **wild-type** | 47.5(19-88) | 84/56 | 8/102/16 | 81(26-145) | 11.5(1-186) | 44(5-621) | 51.6(4-94) | 3/38/45/39/6 |
|  | ***p* value** | 0.455 | 0.002 | 0.523 | 0.262 | 0.004 | 0.193 | 0.057 | 0.407 |
| ***NRAS*** | **mutation** | 37(20-71) | 13/10 | 1/18/3 | 80(49-138) | 23.3(1-167) | 56(6-127) | 46.4(13-90) | 1/3/11/5/1 |
|  | **wild-type** | 48.5(19-88) | 80/68 | 7/107/17 | 81.5(26-145) | 13.6(1-333) | 41(5-621) | 56.2(4-94) | 3/40/49/41/6 |
|  | ***p* value** | 0.067 | 0.825 | 1.000 | 0.582 | 0.637 | 0.332 | 0.229 | 0.334 |
| ***DNMT3A*** | **mutation** | 53(24-76) | 14/19 | 0/28/5 | 76(43-132) | 24.1(2-333) | 60(9-340) | 66(4-85) | 0/3/17/10/1 |
|  | **wild-type** | 45(19-88) | 79/59 | 8/97/15 | 82(26-145) | 11.6(1-208) | 40(5-621) | 53.6(5-94) | 4/40/43/36/6 |
|  | ***p* value** | 0.013 | 0.125 | 0.326 | 0.358 | 0.073 | 0.295 | 0.223 | 0.063 |
| ***TET2*** | **mutation** | 57(28-76) | 11/12 | 0/16/3 | 77(43-132) | 17.1(1-186) | 52(8-167) | 58.6(21-92) | 0/3/13/5/0 |
|  | **wild-type** | 47(19-88) | 82/66 | 8/109/17 | 82(26-145) | 14.05(1-333) | 41(5-621) | 54.3(4-94) | 4/40/47/41/7 |
|  | ***p* value** | 0.102 | 0.497 | 0.694 | 0.102 | 0.205 | 0.122 | 0.591 | 0.204 |
| ***ASXL1*** | **mutation** | 60(20-70) | 11/3 | 0/10/3 | 76.5(47-95) | 2.9(1-167) | 42(6-234) | 35.3(20-94) | 0/3/6/4/0 |
|  | **wild-type** | 47(19-88) | 82/75 | 8/115/17 | 81(26-145) | 18.1(1-333) | 42(5-621) | 55.7(4-94) | 4/40/54/42/7 |
|  | ***p* value** | 0.022 | 0.058 | 0.469 | 0.018 | 0.234 | 0.858 | 0.057 | 0.975 |
| ***CEBPA*** | **double mutation** | 43(25-74) | 14/9 | 0/21/0 | 95(72-141) | 27(3-208) | 23(5-234) | 65.2(20-92) | 2/5/12/3/1 |
|  | **wild-type** | 48(19-88) | 79/69 | 8/104/20 | 79(26-145) | 13.6(1-333) | 44(6-621) | 52(4-94) | 2/38/48/43/6 |
|  | ***p* value** | 0.612 | 0.502 | 0.064 | 0.003 | 0.585 | 0.124 | 0.111 | 0.074 |
| ***RUNX1*** | **mutation** | 53(29-66) | 7/8 | 1/10/3 | 75(59-126) | 5.3(1-174) | 38(15-121) | 47(21-74) | 0/3/6/5/0 |
|  | **wild-type** | 47(19-88) | 86/70 | 7/115/17 | 81(26-145) | 15.6(1-333) | 42(5-621) | 56.2(4-94) | 4/40/54/41/7 |
|  | ***p* value** | 0.038 | 0.530 | 0.408 | 0.391 | 0.404 | 0.440 | 0.066 | 0.939 |

WBC, white blood cell; BM, bone marrow; ITD, internal tandem duplication. a, Two-sample Student’s t-test was used for the evaluations of the difference between groups. b, Chi-squared test was used for the evaluations of the difference between groups. c, Fisher’s exact test was used for the evaluations of the difference between groups.

**Supplementary Table S7** The distribution of mutation numbers within each group stratified by age, FAB subtype and response to chemotherapy.

| Mutation number | Age(years old) | | | FAB subtype | | | Response to chemotherapy | |
| --- | --- | --- | --- | --- | --- | --- | --- | --- |
|  | ≤39 | 40-59 | ≥60 | M2 | M4 | M5 | Sensitive | Refractory |
| 0 | 11 | 7 | 4 | 9 | 0 | 10 | 16 | 1 |
| 1 | 14 | 18 | 5 | 11 | 11 | 11 | 26 | 4 |
| 2 | 18 | 12 | 11 | 11 | 15 | 9 | 29 | 6 |
| 3 | 8 | 18 | 12 | 3 | 19 | 9 | 20 | 5 |
| 4 | 5 | 7 | 6 | 4 | 11 | 1 | 9 | 3 |
| 5 | 3 | 2 | 2 | 2 | 1 | 4 | 5 | 1 |
| 6 | 0 | 5 | 1 | 2 | 3 | 1 | 2 | 2 |
| 7 | 0 | 2 | 0 | 1 | 0 | 1 | 0 | 1 |

**Supplementary Table S8** The numbers of patients in individual groups divided according to response to chemotherapy.

| **Cohort** | **Mutation** | **status** | **CR** | **NR** | ***P* value** |
| --- | --- | --- | --- | --- | --- |
| Total AML | *DNMT3A* | mut | 16 | 9 | 0.019 |
|  |  | wt | 97 | 16 |  |
|  | *FLT3-ITD* | mut | 14 | 9 | 0.008 |
|  |  | wt | 99 | 16 |  |
| IR-AML | *DNMT3A* | mut | 13 | 9 | 0.014 |
|  |  | wt | 70 | 12 |  |
|  | *FLT3-ITD* | mut | 12 | 8 | 0.026 |
|  |  | wt | 71 | 13 |  |
|  | *RUNX1* | mut | 3 | 4 | 0.029 |
|  |  | wt | 80 | 17 |  |
| *De novo* AML | *DNMT3A* | mut | 15 | 9 | 0.013 |
|  |  | wt | 89 | 13 |  |
|  | *FLT3-ITD* | mut | 11 | 9 | 0.002 |
|  |  | wt | 93 | 13 |  |

CR, complete remission; NR, non-remission. Fisher’s exact test was used for each comparison.

**Supplementary Table S9** The insignificant variables in multivariate analysis of survival and their *p* values.

| Variables | *P* value |
| --- | --- |
| OS |  |
| Number of mutations | 0.678 |
| Gender (male *vs* female) | 0.980 |
| Cytogenetics (s-AML *vs de novo* AML) | 0.940 |
| Age (over 60 years old) | 0.094 |
| HDAC (yes *vs* no) | 0.204 |
| HMA (yes *vs* no) | 0.658 |
| *ASXL1* mutation (yes *vs* no) | 0.466 |
| FAB subtype | 0.663 |
| RFS |  |
| Number of mutations | 0.992 |
| Gender (male *vs* female) | 0.111 |
| Cytogenetics (s-AML *vs de novo* AML) | 0.669 |
| Age (over 60 years old) | 0.359 |
| HDAC (yes *vs* no) | 0.937 |
| HMA (yes *vs* no) | 0.686 |
| WBC count (>100×10^9^/L *vs* ≤100×10^9^/L) | 0.077 |
| Number of cycles to CR (≥3 cycles *vs* ≤2 cycles) | 0.449 |
| *ASXL1* mutation (yes *vs* no) | 0.384 |
| *CEBPAdm* (yes *vs* no) | 0.814 |
| *DNMT3A* mutation (yes *vs* no) | 0.102 |
| *FLT3*-ITD mutation (yes *vs* no) | 0.858 |
| *IDH1* mutation (yes *vs* no) | 0.446 |
| *IDH2* mutation (yes *vs* no) | 0.286 |
| *KRAS* mutation (yes *vs* no) | 0.670 |
| *NPM1* mutation (yes *vs* no) | 0.786 |
| *NRAS* mutation (yes *vs* no) | 0.450 |
| *PTPN11* mutation (yes *vs* no) | 0.276 |
| *RUNX1* mutation (yes *vs* no) | 0.841 |
| *SRSF2* mutation (yes *vs* no) | 0.576 |
| *STAG* mutation (yes *vs* no) | 0.282 |
| *TP53* mutation (yes *vs* no) | 0.164 |
| *U2AF1* mutation (yes *vs* no) | 0.112 |
| *WT1* mutation (yes *vs* no) | 0.094 |
| FAB subtype | 0.663 |

OS, overall survival; s-AML, secondary AML; HDAC, high-dose cytosine arabinoside; HMA, hypomethylating agent; RFS, relapse-free survival; WBC, white blood cell; CR, complete remission. COX proportional hazard regression analysis was used for evaluations of each variables.

**Supplementary Table S10** The numbers of patients in aspect of *TET2* mutation and allo-HSCT.

| Survival | Mutations | Status | Allo-HSCT | No allo-HSCT | P value |
| --- | --- | --- | --- | --- | --- |
| RFS | *TET2* | mut | 4 | 8 | 0.210 |
|  |  | wt | 53 | 48 |  |
| OS | *TET2* | mut | 4 | 18 | 0.123 |
|  |  | wt | 53 | 100 |  |

Allo-HSCT, allo-hematopoietic stem cell transplantation; RFS, relapse-free survival; OS, overall survival. Chi-squared test was used for each comparison.

**
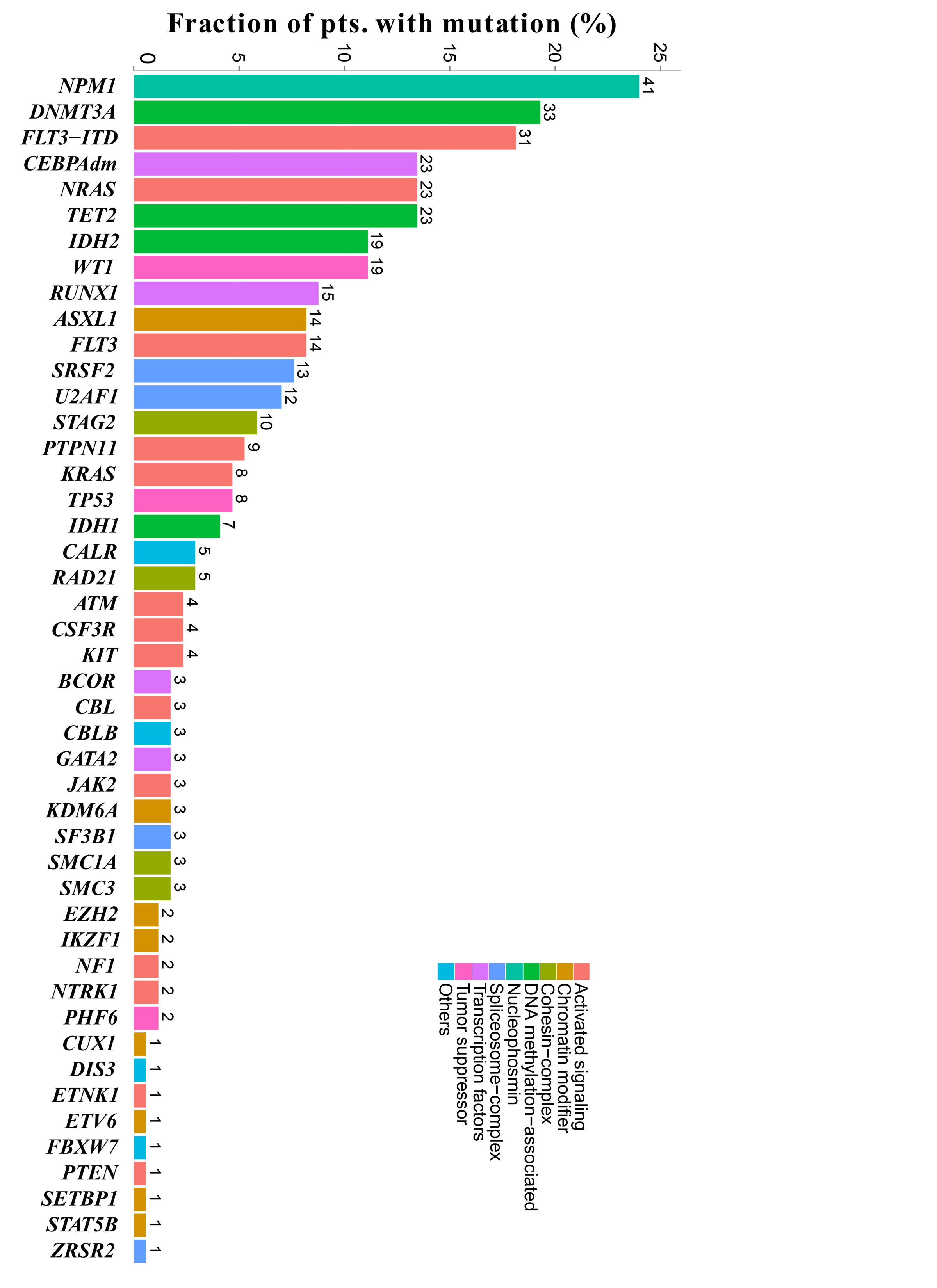
**

**Supplementary Figure S1** Histogram showing the frequency of each genetic mutation. Bars are colored according to the functional cluster assigned to each gene. The number on each bar indicates the number of patients carrying the indicated gene mutation.

**
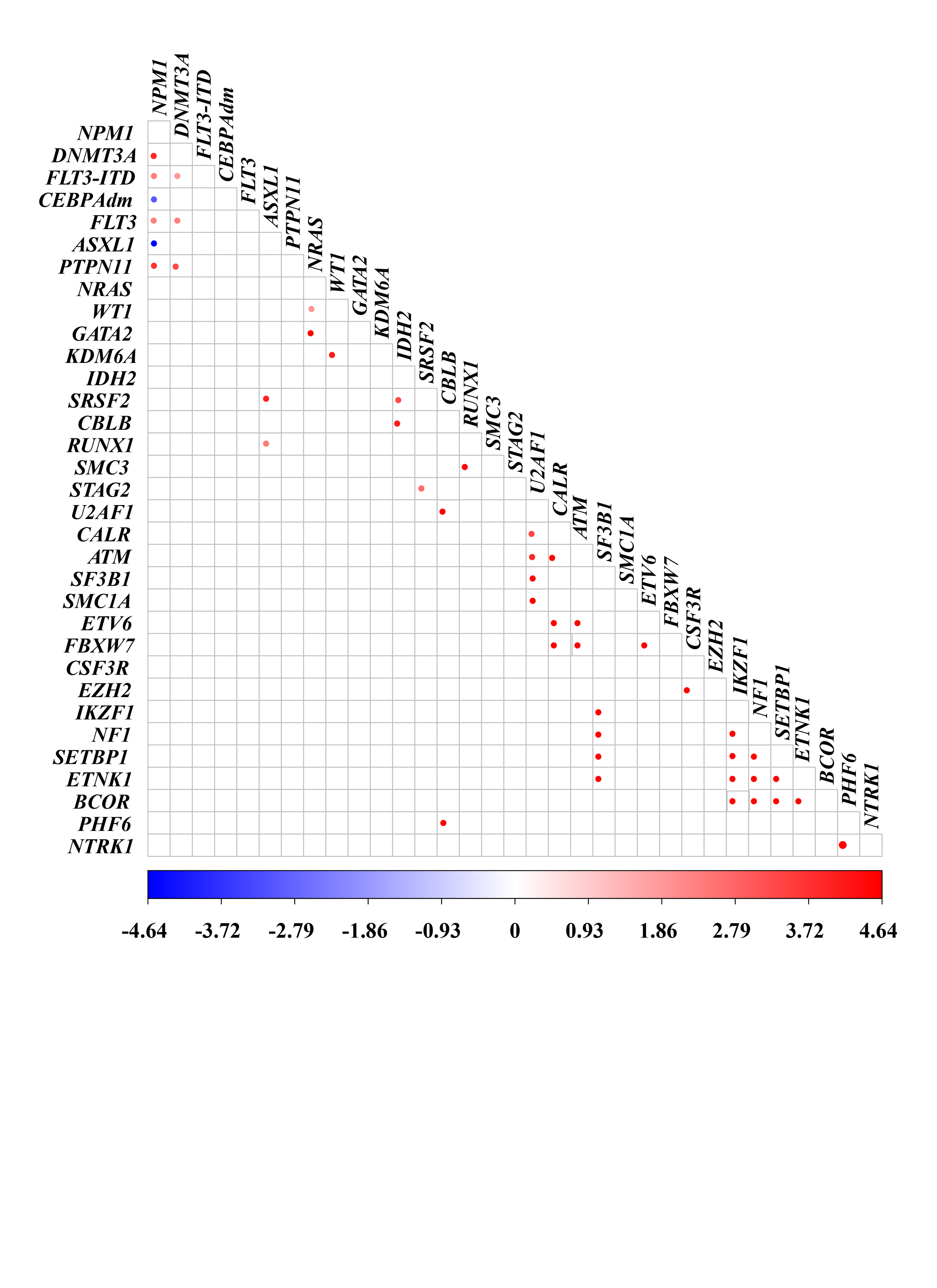
**

**Supplementary Figure S2** Correlation or exclusion of genetic mutation with other alterations. The color of red corresponds to the statistical significance of co-occurrence, while the color of blue corresponds to the statistical significance of mutual exclusion. The deeper the color is, the more significant the association is.

**
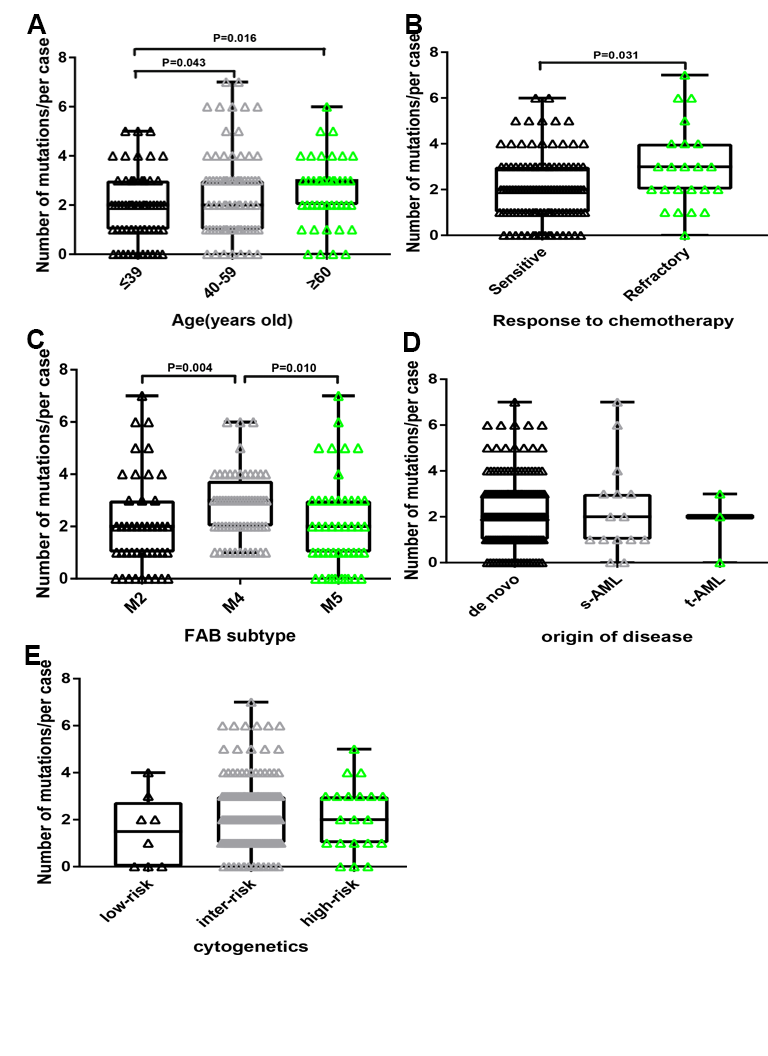
**

**Supplementary Figure S3** The relationship between the number of genetic mutation of each patients and the clinical characteristics including (A) age（adult ≤39, n=59; adult 40-59, n=71; adult ≥60, n=41）, (B) response to chemotherapy（sensitive group, n=107; refractory group, n=23）, (C) FAB subtype（M2, n=43; M4, n=60; M5, n=46）, (D)origin of disease (*de novo*, n=153; s-AML, n=15; t-AML, n=3) and (E)cytogenetics (low-risk, n=8; intermediate-risk, n=125; high-risk, n=20). Center line, median; box limits, upper (75%) and lower (25%) quartiles; whiskers, range from minimum to maximum; triangles in different colors, patients in different subgroups. For each panel, Mann-Whitney *U* test was used to evaluate the differences between each two groups (for example, sensitive group *vs* refractory group, M2 *vs* M4), while Kruskal-Wakkis *H* test was used to evaluate the total differences among three groups (for example, M2 *vs* M4 *vs* M5 subtype).

**Supplementary Figure S4** Kaplan-Meier curve for survival stratified by the status of single genetic mutation in newly diagnosed AML patients. The green and blue lines represent the survival of patients with or without mutations respectively. (A-B) RFS for patients with or without *TET2* mutations in IR-AML (*p*=0.004) and CN-AML (*p <* 0.001). (C) OS for patients with or without *DNMT3A* mutations in CN-AML (*p*=0.024). (D-E) OS for patients with or without *ASXL1* mutations in groups under 60 years of age (*p*=0.015) and IR-AML (*p*=0.025). (F) OS for patients with or without *FLT3*-ITD in groups not receiving all0-HSCT (*p*=0.014). (G-H) OS for patients with or without *NRAS* mutations in IR-AML (*p*=0.014) and CN-AML (*p*=0.011). (I) OS for patients with or without *CEBPAdm* in groups under 60 years of age (*p*=0.041).
